# Intercellular friction and motility drive orientational order in cell monolayers

**DOI:** 10.1073/pnas.2319310121

**Published:** 2024-09-20

**Authors:** Michael Chiang, Austin Hopkins, Benjamin Loewe, M. Cristina Marchetti, Davide Marenduzzo

**Affiliations:** ^a^Scottish Universities Physics Alliance, School of Physics and Astronomy, University of Edinburgh, Edinburgh EH9 3FD, United Kingdom; ^b^Department of Physics, University of California Santa Barbara, Santa Barbara, CA 93106; ^c^Facultad de Física, Pontificia Universidad Católica de Chile, Santiago 7820436, Chile

**Keywords:** intercellular friction, solid–liquid transition, nematic and hexatic order, topological defects, cellular extrusion

## Abstract

Spatiotemporal patterns in multicellular systems are important to understanding tissue dynamics, for instance, during embryonic development and disease. Here, we use a multiphase field model to study numerically the behavior of a near-confluent monolayer of deformable cells with intercellular friction. Varying friction and cell motility drives a solid–liquid transition, and near the transition boundary, we find the emergence of local nematic order of cell deformation driven by shear-aligning cellular flows. Intercellular friction contributes to the monolayer’s viscosity, which significantly increases the spatial correlation in the flow and, concomitantly, the extent of nematic order. We also show that local hexatic and nematic order are tightly coupled and propose a mechanical-geometric model for the colocalization of +1/2 nematic defects and 5–7 disclination pairs, which are the structural defects in the hexatic phase. Such topological defects coincide with regions of high cell–cell overlap, suggesting that they may mediate cellular extrusion from the monolayer, as found experimentally. Our results delineate a mechanical basis for the recent observation of nematic and hexatic order in multicellular collectives in experiments and simulations and pinpoint a generic pathway to couple topological and physical effects in these systems.

## Significance

Understanding the mechanisms underlying pattern formation and flow in biological tissues is important in embryology and disease. An enigmatic observation in this field is the spontaneous emergence of nematic order in monolayers of nearly isotropic cells. Here, we propose a mechanism for the simultaneous origin of both local nematic and hexatic order. We show that the interplay between cell motility and intercellular friction yields macroscopic spatiotemporal correlations in multicellular flow, which deform and align cells. Our simulations also reveal that cellular geometry implies a fundamental relation between hexatic and nematic defects, which colocalize with cell–cell overlaps and may create hotspots for cellular extrusion.

The collective migration of cells within a biological tissue plays a fundamental role in physiological and pathological processes such as embryogenesis (1), wound healing (2), and cancer progression (3, 4). A long-standing challenge is to dissect the molecular mechanisms driving such coordinated motion—from a biophysical standpoint, one aims to understand how the mechanical properties of individual cells and the forces acting upon them give rise to the emergent phenomena seen at the tissue scale (5).

Many biological processes, such as tissue development and cancer metastasis, involve a change in cell collective dynamics between a solid-like and a liquid-like state, and have been compared to rigidity and jamming transitions in other soft matter systems (6, 7). Modeling has provided fruitful insight into the mechanisms driving these transitions (8, 9). For instance, vertex (10), self-propelled Voronoi (11, 12), cellular Potts (13, 14), and multiphase field models (15, 16) have successfully captured several defining features of tissue jamming–unjamming, showing how cell shape, deformability, and cell–cell adhesion are some of the key determinants of this transition.

Apart from the change in fluidity, another collective phenomenon of tissue monolayers is the spontaneous emergence of orientational nematic order. This has been recently observed in both experiments and simulations (17–25), where nematic order has been measured in terms of the cell shape orientation. Yet, the physical mechanism underlying the origin of cell alignment is still elusive. For example, it is unclear whether contractile or extensile activity is required to create nematic order, nor has it been discussed how robust the order is across the parameter space. The latter aspect is important as, for instance, cells in epithelial tissue in the solid or glass phase are quite isotropic, so any order is by necessity linked with relatively small shape fluctuations. Additionally, as these systems also exhibit bond-orientational (hexatic) order (12, 14, 15, 25), it is important to understand how nematic and hexatic order interact with each other, and how pervasive the proposed combined “hexanematic” order (26, 27) is in practice.

In this work, we study the dynamics and phase behavior of a confluent monolayer of deformable and motile cells with intercellular and cell–substrate friction. We observe a solid–liquid transition that is accompanied by the emergence of both local hexatic and nematic order. This orientational order is maximal close to the transition and is driven by the onset of a cellular flow, which deforms and aligns cells within a range set by the correlation length of the flow. Moreover, we find that the geometry of our deformable cells creates a coupling between the hexatic and nematic order, such that defects in the hexatic order, corresponding to 5–7 disclination pairs, appear preferentially close to +1/2 defects in the nematic order. These defects correlate with regions of enhanced overlap between cells, which provides a mechanism for the topological creation of hotspots for cellular extrusion, as found experimentally (17) and in simulations (15, 24). Our results therefore provide a mechanical explanation for the emergence of nematic order in cell monolayers and predict that the latter should be strongly enhanced by intercellular friction. Our finding of an intimate geometric coupling between the hexatic and nematic order provides a mechanical underpinning for the proposal that cellular monolayers can behave, under suitable conditions, as a hexanematic fluid (26, 27). They also raise the tantalizing possibility that topology can play an important role in multicellular dynamics and developmental biology.

## Results

### A Multiphase Field Model for Deformable Cell Monolayers with Cell–Cell and Cell–Substrate Friction.

**Fig. 1.**
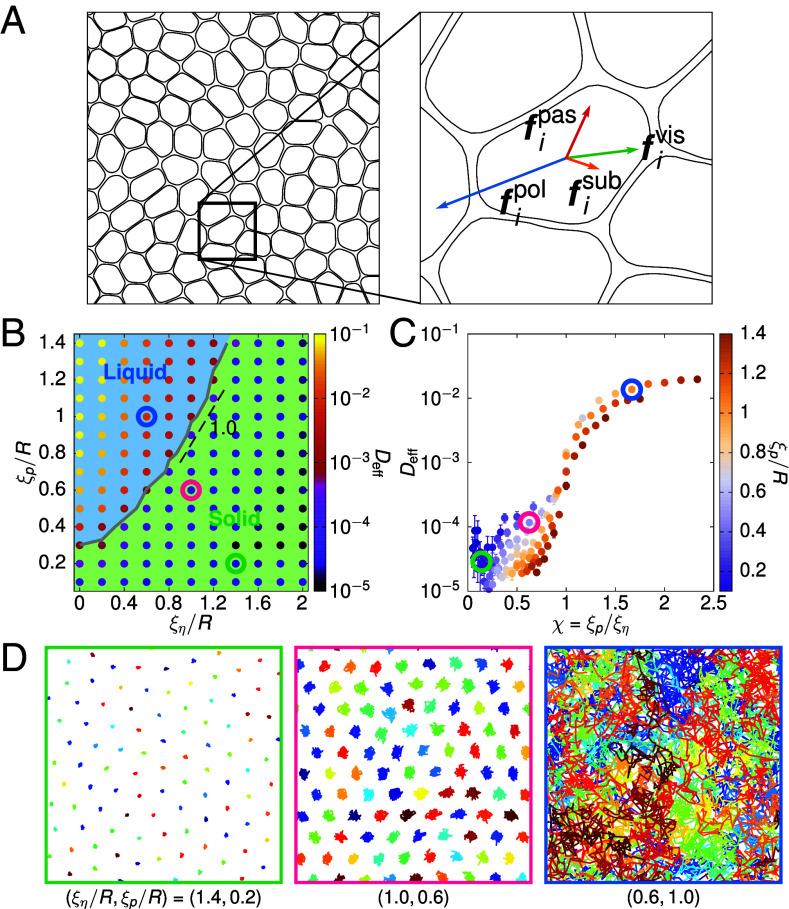
Intercellular friction and motility drive a solid-liquid transition in a multiphase field model. (*A*) A snapshot of the simulated monolayer (*Left*) with an enlarged view of one of the cells showing the forces acting upon it (*Right*). There are four types of forces: a passive force $f_{i}^{\text{pas}}$ due to the imposed free energy, an active self-propulsion force $f_{i}^{\text{pol}}$, cell–substrate friction $f_{i}^{\text{sub}}$, and cell–cell friction $f_{i}^{\text{vis}}$. (*B*) Phase diagram showing the effective diffusivity $D_{\text{eff}}$ of the system when varying cell motility $\xi_{p}$ and the flow screening length $\xi_{\eta }$ (in units of the cell radius R). The transition line demarcating the solid and fluid phases is interpolated based on the threshold $D_{\text{eff}}=10^{-3}$. (*C*) Points where $\xi_{\eta }/R\geq 0.6$ collapse onto a master curve as a function of the ratio between the persistence and flow screening lengths, $\chi =\xi_{p}/\xi_{\eta }$. (*D*) Representative cell trajectories at three points of the phase diagram [corresponding to those circled in (*B* and *C*)], indicating the transition from solid-like (caging) to fluid-like (neighbor exchange) behavior.

We simulate N cells as deformable droplets randomly initialized on a substrate (Fig. 1*A*). Each cell is modeled by a phase field $\phi_{i}\left(r\right)$ (i=1,…,N), where $\phi_{i}=1$ marks the cell’s interior and 0 its exterior. In line with previous work (15, 16, 20, 23, 28, 29), the droplet’s shape is controlled by a Landau–Ginzburg-like free energy (*Materials and Methods*), which includes Cahn–Hilliard terms governing the cell’s edge tension σ and thickness ξ, a soft constraint on its area (with radius R), and a repulsive term (strength ϵ with units $\left[E\right]{\left[L\right]}^{-2}$) that minimizes cell overlaps. Each cell experiences a passive force $f_{i}^{\text{pas}}$ due to the imposed free energy (30) and an active self-propulsion force $f_{i}^{\text{pol}}$ (proportional to speed $v_{0}$) as it undergoes rotational diffusion with rate $D_{r}$. Importantly, these forces are balanced by cell–cell friction $f_{i}^{\text{vis}}$ (strength η with units $\left[E\right]\left[T\right]{\left[L\right]}^{-2}$) due to the relative motion between cells and cell–substrate friction $f_{i}^{\text{sub}}$ (strength Γ with units $\left[E\right]\left[T\right]{\left[L\right]}^{-4}$; see Fig. 1*A* and *Materials and Methods* for the expressions of these forces). The droplets are evolved over time using advective-relaxational dynamics, and the net effect of the forces on the droplet enters the equations of motion through its advection velocity $v_{i}$.

The overall behavior of the model is tuned by three parameters. First, there is the deformability of individual droplets, d≡ϵξR/(12σR), where at lower d cells remain circular and prefer to overlap rather than deform (15, 28, 29). Here, we set d=4.16 such that cells are highly compliant and do not overlap significantly. Second, the cell’s motility is tuned by the Péclet number $\text{Pe}=\xi_{p}/R$, where $\xi_{p}=v_{0}/D_{r}$ is the persistence length of cell motion. Third, the relative strength of cell–cell to cell–substrate friction gives a flow screening length $\xi_{\eta }=\sqrt{\eta /\Gamma }$ (31–33) that quantifies the distance over which the motion of a cell can influence that of another. Throughout this work, we focus on varying $\xi_{p}$ and $\xi_{\eta }$ to observe their effect on the monolayer dynamics; in particular, we fix cell–substrate friction Γ and vary the strength of cell–cell friction η. We use a system of N=100 and, in selected cases, 400 cells—results for the latter are shown in *SI Appendix* unless otherwise stated. We estimate the physical values of the simulation parameters by mapping to data on mammary epithelial MCF-10A cells (34, 35) (*SI Appendix*), and we find comparable values for the persistence length of motility and velocity correlations between simulations and experiments.

### Interplay of Friction and Cell Motility Drives a Solid–Liquid Transition in the Monolayer.

We first examine the role of cell motility ($\xi_{p}$) and of the two friction forces ($\xi_{\eta }$) on tissue fluidity. To this end, we measure the mean square displacement (MSD) of individual cells and compute an effective diffusivity $D_{\text{eff}}=\;\lim_{t\to \infty }\text{MSD}/\left(4D_{0}t\right)$, where $D_{0}=v_{0}^{2}/\left(2D_{r}\right)$ is the self-diffusivity of an isolated active Brownian particle (15). Fig. 1*B* displays a phase diagram of $D_{\text{eff}}$ and shows that, above a critical $\xi_{p}$, the monolayer undergoes a solid–liquid transition, where cells move from being caged to exchanging neighbors (see Fig. 1*D* and also *SI Appendix*, Fig. S2 for N=400).

We construct the transition line using the threshold $D_{\text{eff}}=10^{-3}$, which aligns well with changes in the structural order between the two phases as presented below. The line shows that as $\xi_{\eta }$ increases, or when cell–cell friction dominates over cell–substrate friction, a higher $\xi_{p}$ is required for the monolayer to melt. Notably, it becomes almost linear when $\xi_{\eta }\geq 0.6$, but is strongly nonlinear below this point (Fig. 1*B*), suggesting there are different mechanisms regulating the melting process. At high $\xi_{\eta }$, the linearity implies that the transition can be described by a single dimensionless parameter $\chi =\xi_{p}/\xi_{\eta }$, which quantifies the competition of two length scales—the persistence length of a cell’s active propulsion and the correlation length of the motion between cells. Using this definition, points on the phase diagram, at least when $\xi_{\eta }\geq 0.6$, all collapse onto a master curve, and the melting takes place when χ∼1 (Fig. 1*C* and *SI Appendix*, Fig. S2). Note that χ fails to capture the transition accurately when $\xi_{\eta }<0.6$. Here, the transition line flattens as $\xi_{\eta }\to 0$, suggesting that there is a regime where melting is independent of $\xi_{\eta }$, and other mechanisms, such as cell deformability (15), may have a larger effect in melting the system.

### A Local Hexatic Regime Separates the Liquid and Glassy Solid Phases.

**Fig. 2.**
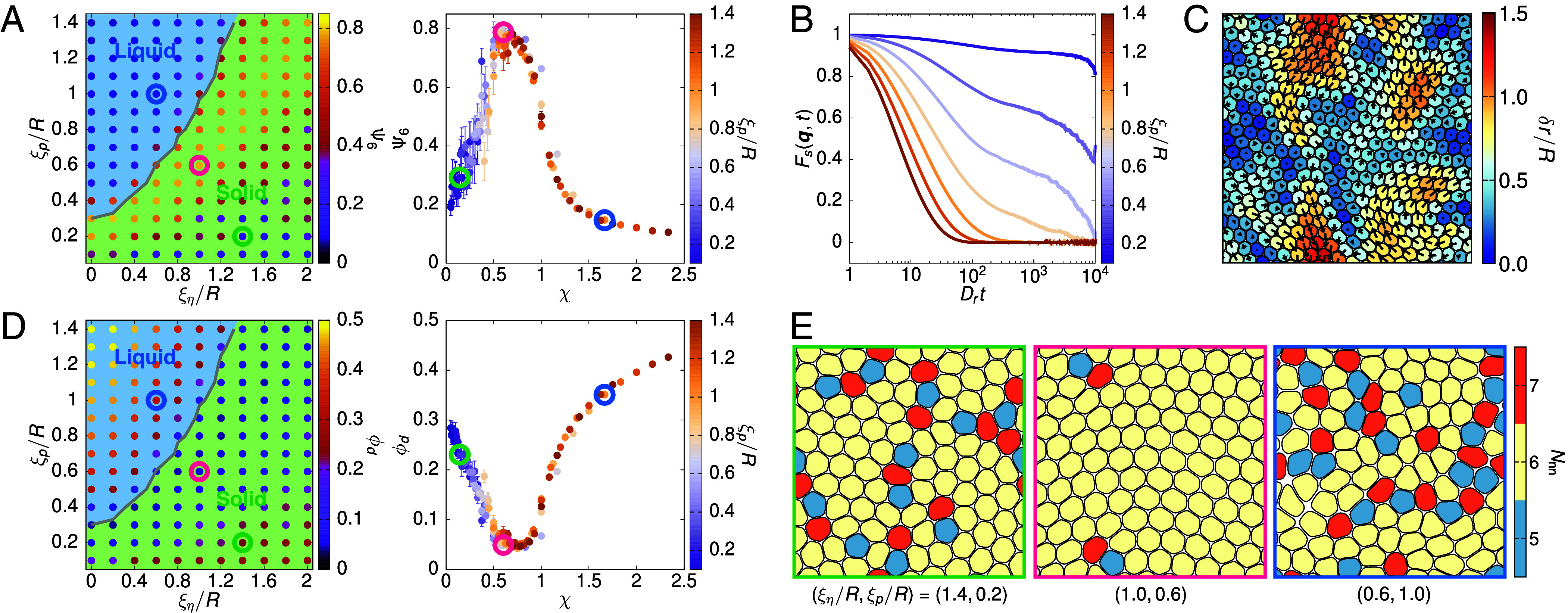
The monolayer exhibits a regime with local hexatic order between a liquid and a glassy, amorphous solid phase. (*A*) *Left*: Measurements of the global bond-orientational order $\Psi_{6}$ superposed on the solid–liquid phase diagram shown in Fig. 1*B*. *Right*: Collapsing $\Psi_{6}$ based on χ onto a master curve for points where $\xi_{\eta }\geq 0.6$. (*B*) Self-intermediate scattering function $F_{s}\left(q,t\right)$ (with $\left|q\right|=\pi /R$) when varying χ (i.e., vary $\xi_{p}$ at fixed $\xi_{\eta }=1.0$) for a system of N=400 cells. (*C*) Cell displacement map of a 400-cell monolayer at $\xi_{\eta }/R=1.0$ and $\xi_{p}/R=0.6$ over a period of time $D_{r}t=100$, corresponding to the timescale when $F_{s}\left(q,t\right)$ decays to ∼1/2. Arrows indicate the directions of the cell displacements, with their length twice the magnitude of the actual displacements to aid visualization. (*D*) Similar to (*A*), but showing the average fraction of cells with disclinations $\phi_{d}$ in the monolayer. (*E*) Representative snapshots of the system at parameter points circled in the phase diagrams in (*A* and *D*), with cells colored by their number of nearest neighbors $N_{\text{nn}}$, determined by Voronoi tessellation. Here, fivefold and sevenfold disclinations are marked in blue and orange, respectively.

To characterize the structural order of the system, we compute for each cell its bond-orientational order $\psi_{6,j}=\frac{1}{N_{j,\text{nn}}}\sum_{k\in \text{nn}}e^{i6\theta_{\mathrm{jk}}}$, where the sum is over the nearest neighbors and $\theta_{\mathrm{jk}}$ is the angle between the x-axis and the bond vector linking cells j and k. The quantity $\Psi_{6}=\langle \left|\frac{1}{N}\sum_{j=1}^{N}\psi_{6,j}\right|\rangle$ then gives the global orientational order of the monolayer. As the system solidifies, $\Psi_{6}$ first increases to near unity, indicating high order, before it surprisingly decreases to a low value deep in the solid, indicating that the transition is distinct from conventional two-dimensional (2D) melting where the hexatic phase is a liquid (Fig. 2*A* and *SI Appendix*, Fig. S7*B*). The weakening of $\Psi_{6}$ deep in the solid regime can be attributed to the random positioning of cells when initializing the monolayer and indicates that the solid regime is a glass, as supported by the behavior of the self-intermediate scattering function and the non-Gaussian parameter, as well as maps of cell displacements (Fig. 2 *B* and *C* and *SI Appendix*, Figs. S4 and S5). These maps show correlated regions that extend over multiple cell widths and are bound by regions of high strain, as commonly seen in experiments on epithelia. The long relaxational timescale associated with glassy dynamics means that the monolayer remains kinetically frozen in its initial state and is unable to reach a crystalline configuration with lower free energy. Indeed, by starting the simulations with cells arranged on a triangular lattice, $\Psi_{6}$ approaches unity asymptotically as the system freezes (*SI Appendix*, Fig. S6*B*). In line with this, the global translational order $\Psi_{T}$ of the system (*SI Appendix*) only changes significantly across the solid–liquid transition when cells are initialized on a lattice (*SI Appendix*, Figs. S3, S6*C*, and S7*C*).

Since the spatial organization of structural defects, i.e., fivefold and sevenfold disclinations, plays a prominent role in 2D melting (14, 15, 36–40), we monitor their presence within our model tissue by counting the number of nearest neighbors $N_{\text{nn}}$ of each cell (Fig. 2*E* and *SI Appendix*, Figs. S6*A* and S7*A*). Consistent with $\Psi_{6}$, the fraction of cells with disclinations $\phi_{d}$ first decreases as the monolayer freezes but increases again further within the solid regime (Fig. 2*D* and *SI Appendix*, Fig. S7*D*). We find fivefold and sevenfold disclinations are bound in pairs in the intermediate regime of local hexatic order and become unbound in the liquid phase. Some isolated disclinations are seen in the solid phase due to slow, glassy dynamics in relaxing from the initial conditions. While the patterning of defects may appear similar in the glassy solid and liquid phases, the dynamics is different: In the solid regime, $\phi_{d}$ is static as disclinations are pinned to the same cells, whereas in the liquid, $\phi_{d}$ fluctuates due to binding-unbinding events (*SI Appendix*, Fig. S8 and Movies S1–S3).

### Cellular Flow Promotes Cell Deformation and Local Nematic Order.

Another topological feature that has attracted lots of interest in tissue sheets is the emergence of nematic order in cell deformation, and the accompanying defects have been implicated in various physiological and pathological processes (17, 21, 22, 41). While previous work (16, 17, 20, 23, 42) has mostly focused on how active stresses, such as individual cell contractility, drive nematic order, here we demonstrate an alternate mechanism by showing that the interplay between intercellular friction and motility can spontaneously give rise to local nematic alignment. In line with recent studies (16, 20, 23, 28), we use the shape tensor $S_{i}=-\int d^{2}r\;\left(\nabla \phi_{i}\right){\left(\nabla \phi_{i}\right)}^{T}$ to determine the cell deformation axis (i.e., the eigenvector corresponding to the largest eigenvalue of $S_{i}$) and define a local nematic order parameter $\Psi_{2}^{L}=\langle \left|\frac{1}{N}\sum_{j=1}^{N}\psi_{2,j}\right|\rangle$, where $\psi_{2,j}=\frac{1}{N_{j,\text{nn}}}\sum_{k\in \text{nn}}e^{i2\theta_{\mathrm{jk}}^{d}}$ and $\theta_{\mathrm{jk}}^{d}$ is the angle between the deformation axes of cells j and k. In this way, a higher $\Psi_{2}^{L}$ signifies stronger nematic alignment between neighboring cells’ deformation axes.

**Fig. 3.**
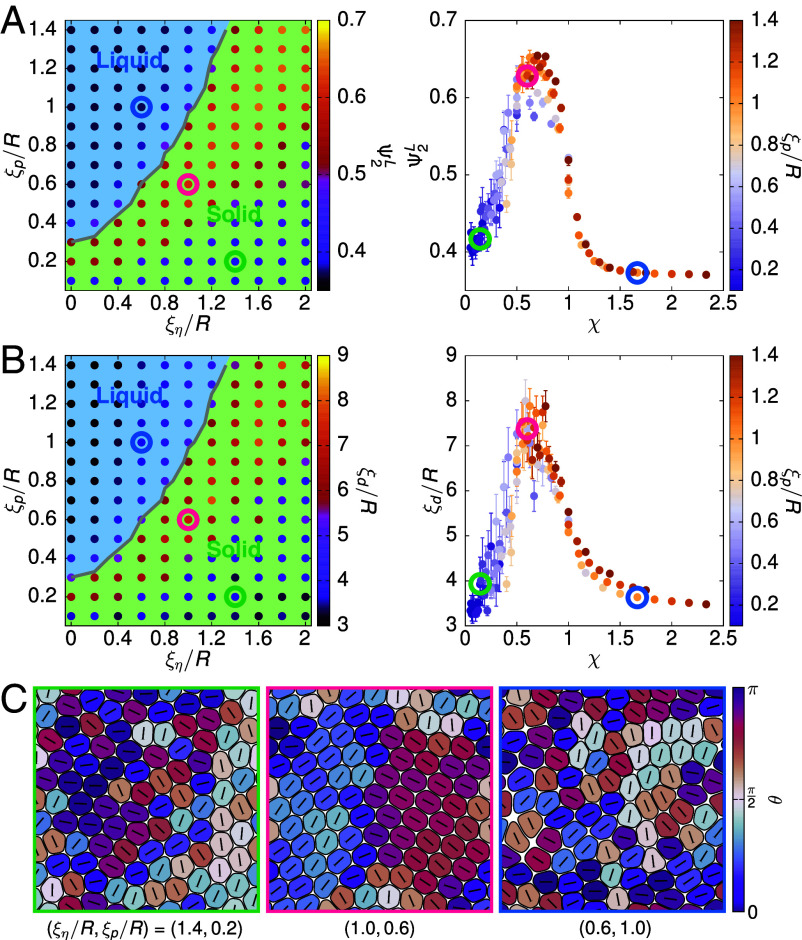
Cell–cell friction drives local nematic alignment of cells. (*A*) *Left*: Measurements of the local nematic order parameter $\Psi_{2}^{L}$ superposed on the phase diagram shown in Fig. 1*B*. *Right*: Collapsing $\Psi_{2}^{L}$ based on χ onto a master curve for points where $\xi_{\eta }\geq 0.6$. (*B*) Similar to (*A*), but for the local characteristic length $\xi_{d}$ of domains in which cells exhibit coherent nematic alignment. (*C*) Representative snapshots of the system at points circled in the phase diagrams in (*A* and *B*), with cells colored by the angle θ between their deformation axis (the line within each cell) and the x-axis.

Remarkably, we find the monolayer exhibits an increase in nematic order in the vicinity of the solid–liquid transition, coinciding with the region of the phase space where there is large hexatic order (Fig. 3*A*). This is visually apparent by coloring cells according to their deformation direction, which shows locally aligned domains (Fig. 3*C* and Movies S4–S6). To quantify domain size, we measure the nematic order within a circle of radius r, averaged across a set of grid points, and then extract a characteristic length $\xi_{d}$ from the decay in the order as a function of r (*SI Appendix*, Fig. S9). This shows that local alignment can be up to four cell lengths (Fig. 3*B*). These results are largely unaffected by the initial conditions and the system sizes (*SI Appendix*, Figs. S10 and S11).

The emergence of local nematic alignment near the solid–liquid transition can be rationalized by the following argument. First, cellular flow can lead to both cell deformation and cell alignment (43). In a coarse-grained model, the rate of change of the deformation tensor should be proportional to the local shear rate, which we can estimate as $∼v_{0}/\xi_{\eta }$, with $v_{0}$ a typical velocity scale. Dimensional analysis then suggests that the flow-induced cell deformation and local nematic order should increase as $∼v_{0}/\left(\xi_{\eta }D_{r}\right)=\chi$ (*SI Appendix*, Fig. S12*A*). Importantly, this local order can only persist up to the correlation length of the flow, which is proportional to $\xi_{\eta }$ (*SI Appendix*, Fig. S12*B*); as a result, the order decreases when the cell persistence length $\xi_{p}$ becomes larger than $\xi_{\eta }$ (i.e., χ>1), as cells move away before aligning. These two opposing effects on the local nematic order when increasing χ therefore argue that the order should be maximal when χ∼1, in line with our simulation results.

### Structural Disclinations Determine the Location of Nematic Defects and Hotspots for Cellular Extrusion.

**Fig. 4.**
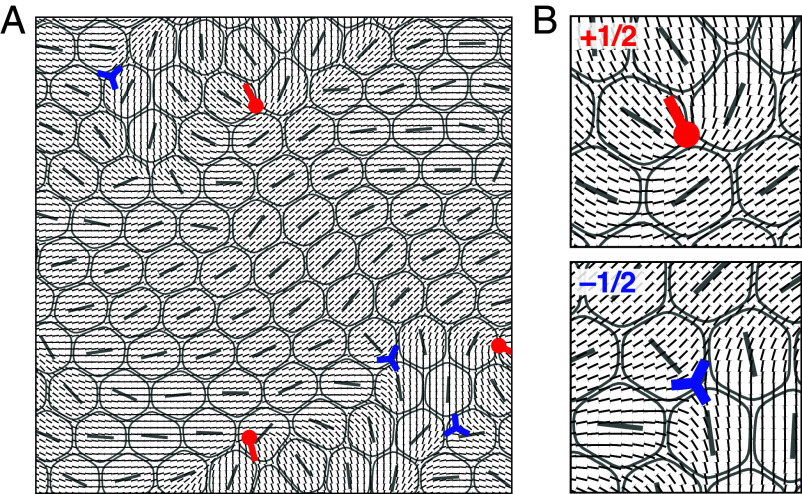
+1/2 and −1/2 defects emerge near boundaries of local nematic domains. (*A*) A simulation snapshot showing the deformation axes of individual cells, the coarse-grained director field derived from the Q tensor (*SI Appendix*), and the nematic defects (red tadpoles for +1/2 and blue three-edge stars for −1/2). (*B*) An enlarged view of the director field around a +1/2 and a −1/2 defect.

We next explore the organization of topological defects in the nematic texture, within the region where $\Psi_{2}^{L}$ is large. To this end, we construct a tensor field $Q\left(r\right)=\sum_{i=1}^{N}W_{i}\left(r\right)\;S_{i}\left(2n_{i}n_{i}^{T}-I\right)$, where $S_{i}$ is the degree of deformation of cell i, $n_{i}$ is its deformation axis, and $W_{i}\left(r\right)$ is an ellipsoidal smoothing function (*SI Appendix*). Nematic defects are then identified by finding local minima in the scalar component of Q and computing the topological charge. As shown in Fig. 4, ±1/2 defects arise near the boundaries of local nematic domains.

**Fig. 5.**
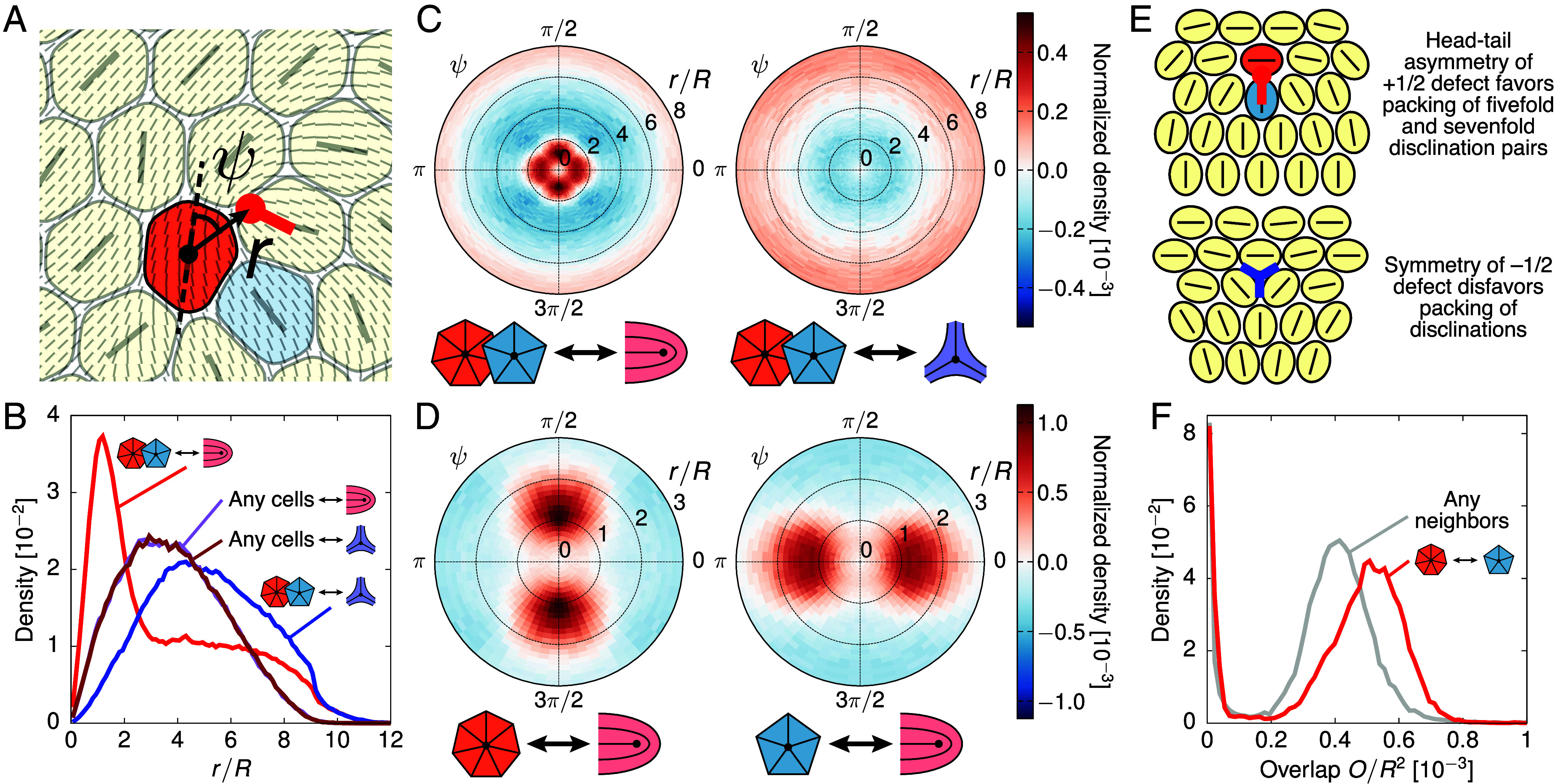
Hexatic 5–7 disclination pairs are strongly correlated with nematic defects. (*A*) A simulation snapshot illustrating the radial and angular observables (r,ψ) measured when relating a nematic defect (red tadpole) to its closest hexatic disclination (orange cell). Here, r is the distance between the two defects and ψ is the angle that the cell deformation axis of the hexatic disclination makes with the vector connecting it to the nematic defect core. (*B*) Probability density functions (PDFs) of the distance r between ±1/2 nematic defects and their closest hexatic disclination (fivefold or sevenfold), and between ±1/2 defects and a randomly selected cell. (*C*) Polar heatmaps showing the joint PDFs of r and ψ for the relation between hexatic disclinations and (*Left*) +1/2 defects or (*Right*) −1/2 defects. The PDFs are normalized by subtracting the PDF for the relation between a randomly selected cell and the corresponding defect type. (*D*) Similar to (*C*), but focusing only on the relation between +1/2 defects and (*Left*) sevenfold or (*Right*) fivefold disclinations. (*E*) Schematics explaining how the geometric layout of the comet-like +1/2 nematic defect favors colocalization with 5–7 pairs, whereas the −1/2 defect does not. (*F*) PDFs of the degree of pairwise cell overlap $O_{\mathrm{ij}}=\int d^{2}r\;\phi_{i}^{2}\phi_{j}^{2}$ for a random pair of nearest-neighbor cells (gray curve) and a 5–7 pair (red curve). Results shown here are for the point $\left(\xi_{\eta }/R,\xi_{p}/R\right)=\left(1.0,0.6\right)$.

Recent studies (26, 27) have suggested that a tissue monolayer can simultaneously exhibit both hexatic and nematic orientational order, consistent with our results presented above. Here, we aim to establish the physical connection between the topological defects associated with these two types of order—namely nematic ±1/2 defects and 5–7 disclination pairs, which correspond to dislocations and are the structural defects in the hexatic phase. We first investigate positional correlations between 5–7 pairs and ±1/2 nematic defect. Surprisingly, we find that +1/2 defects are on average significantly closer to 5–7 pairs than −1/2 to these pairs (Fig. 5 *A* and *B*). The latter are also further away from 5–7 pairs than from a randomly chosen cell within the monolayer.

To gain insight into the mechanisms that drive +1/2 defects to form close to 5–7 pairs, we analyze the angular distribution of the relative position of hexatic and nematic defects (Fig. 5 *A* and *C*). While −1/2 defects are isotropically depleted around 5–7 pairs, the angular distribution of +1/2 around 5–7 pairs is anisotropic, with a marked fourfold symmetry (Fig. 5*C*). A separate analysis of fivefold and sevenfold disclinations shows that they contribute complementary parts of the angular distribution pattern (Fig. 5*D*). In particular, the deformation axis of a cell with sevenfold disclination is typically perpendicular to the symmetric axis of a +1/2 defect, whereas the deformation axis of a sevenfold disclination is often parallel with it.

Close inspection of simulation snapshots and movies suggests that the difference in the hexatic structure close to +1/2 and −1/2 defects can be explained geometrically (Fig. 5*E* and Movie S7). The head–tail comet asymmetry of the +1/2 defect, and its corresponding polar nature, is compatible with cell arrangements that readily accommodate a 5–7 pair. In stark contrast, the threefold symmetry of a −1/2 defect is better placed in a region with regular hexagonal packing of cells without structural defects.

This geometric reasoning leads to the expectation that +1/2 defects should be associated with larger cell deformations. Previous experimental work (17) found that +1/2 defects tend to colocalize with regions of high elastic stress and are candidate sites for cellular extrusion from the monolayer. As cell deformation likely correlates with elastic stress, our results suggest a mechanical model for the selection of extrusion hotspots that is driven by the presence of structural 5–7 dislocations, which are attracted by +1/2 nematic defects. Accordingly, a quantitative analysis of our multiphase patterns shows that 5–7 dislocations (and hence +1/2 defects) are associated with increased cell–cell overlaps (Fig. 5*F*), which likely correlate with potential extrusion sites. These results extend previous work showing that extrusion correlates with the location of fivefold disclinations (15, 24), providing a link between topological defects in hexatic and nematic texture.

## Discussions

In summary, we have used multiphase simulations to study the dynamics and topological structure of a monolayer of motile cells with intercellular friction. When the latter is sufficiently large, a key dimensionless parameter for determining the physical properties of the monolayer is the ratio χ between the persistence length—which measures the distance traveled by a cell in isolation before rotational diffusion kicks in—and the flow screening length—which measures the correlation length of cellular flow. Increasing χ triggers a solid–liquid transition (10, 13, 15), which can be clearly identified by measuring the effective diffusion coefficients of the cells.

An important outcome of our investigation is that an orientationally ordered phase emerges close to the solid–liquid transition. This phase has both local hexatic and nematic order—the emergence of the latter is striking given the fact that cells are nearly isotropic in isolation. The appearance of nematic order is due to the onset of correlated cellular flow in the monolayer, as shear forces deform the cells and align them locally (43), within a length scale given by the screening length. We note that the tissue viscosity associated with the cell–cell friction employed in our model can partly come from cell–cell adhesion, and recent experiments have indicated that adhesion plays an important role in determining local hexatic and nematic order (27). The necessity of both cellular flow and a finite flow correlation length explains why nematic order also emerges naturally in monolayers without intercellular friction but with active dipolar forces—e.g., from the contractile actomyosin cortex. In this case, dipolar activity and motility create flow, while activity and elasticity yield a finite correlation length (44) that determines the size of nematic domains.

Our finding of local orientational order near the solid–liquid transition provides mechanical insight into the observation of a local hexanematic phase in cell monolayers in experiments and simulations (26, 27). In particular, we find that geometry leads to an intimate coupling between hexatic and nematic order in the context of topological defects. The shape of cells near comet-like +1/2 favors the presence of 5–7 pairs (i.e., structural dislocations), which are the signature of hexatic order; as a result, 5–7 pairs appear near +1/2 defects. Previous work (45–47) on passive liquid crystals has examined the possible phases in systems with two types of orientational order coupled to each other through suitable terms in a Landau free energy. It would be interesting to ask whether the correlation between nematic defects and 5–7 disclinations reported here can also be understood as the result of an energetic coupling between hexatic and nematic order, and would therefore similarly arise in passive systems.

Finally, the regions with +1/2 defects and 5–7 pairs are associated with both increased cell deformation and cell–cell overlap. We therefore suggest that topological patterns provide a mechanism to select these regions as potential sites for cell extrusion from the monolayer, in agreement both with experimental observations that +1/2 defects in monolayers are extrusion hotspots (17) and with the prediction by simulations that extrusion should occur near fivefold disclinations in the hexatic order (15, 24). We hope our prediction of a geometric coupling between nematic and hexatic defects will stimulate further analysis of the interplay between topology and extrusion in monolayers and model tissues.

## Materials and Methods

### Model Setup.

We consider a 2D monolayer of N deformable cells, each modeled by a phase field $\phi_{i}$ (i=1,⋯,N) with periodic boundary conditions. These cells form a monolayer that is nearly confluent, with a packing fraction of ∼0.95. Similarly to previous work (15, 28, 29), the system’s total free energy is given by

[1] $$\begin{array}{cc} F & =\sum_{i=1}^{N}[\int d^{2}r\;\kappa \left[\phi_{i}^{2}{\left(\phi_{i}-1\right)}^{2}+\xi^{2}{\left(\nabla \phi_{i}\right)}^{2}\right]\\ & \;+\lambda A_{0}{\left(1-\int d^{2}r\frac{\phi_{i}^{2}}{A_{0}}\right)}^{2}+\epsilon \sum_{i<j=1}^{N}\int d^{2}r\;\phi_{i}^{2}\phi_{j}^{2}]\;. \end{array}$$

Here, the first two terms fix $\phi_{i}$ to be close to 1 within the cell and 0 otherwise, with ξ controlling the cell’s interfacial thickness and κ its edge tension σ=κξ/3. The third term constrains the cell area to be near $A_{0}=\pi R^{2}$, where R is the ideal cell radius, and the last term enforces steric repulsion between cells by penalizing overlap. Unless otherwise stated, cells are initially positioned randomly as circular droplets with radius R, and the phase fields are evolved over time using advective-relaxational dynamics

[2] $$\begin{array}{c} \partial_{t}\phi_{i}+\phi_{i}=-\mu_{i}\;, \end{array}$$

where $\mu_{i}=\delta F/\delta \phi_{i}$ is a chemical potential and $v_{i}$ the advection velocity of a cell.

We assume the dynamics to be overdamped and determine $v_{i}$ through a force balance equation that includes four types of forces:

[3] $$\begin{array}{c} f_{i}^{\text{pas}}+f_{i}^{\text{pol}}+f_{i}^{\text{sub}}+f_{i}^{\text{vis}}=0. \end{array}$$

Specifically, there is a passive force

[4] $$\begin{array}{c} f_{i}^{\text{pas}}=-\sum_{j=1}^{N}\int d^{2}r\;\phi_{i}\phi_{j}\nabla \mu_{j} \end{array}$$

as a result of the imposed free energy F. There is a self-propulsion force that accounts for the cell’s motility,

[5] $$\begin{array}{c} f_{i}^{\text{pol}}=\Gamma v_{0}\int d^{2}r\;\phi_{i}P\left(r\right)\;, \end{array}$$

where Γ is a damping constant due to the substrate and $P\left(r\right)=\Phi^{-1}\sum_{j=1}^{N}\phi_{j}p_{j}$ is the tissue polarization (48). Here, $\Phi =\sum_{k=1}^{N}\phi_{k}$ and $p_{j}=\left(\cos\theta_{j},\sin\theta_{j}\right)$ is the cell’s propulsion direction, which is assumed to undergo rotational diffusion with rate $D_{r}$ [i.e., $d\theta_{j}=\sqrt{2D_{r}}\;dW_{j}\left(t\right)$, where $W_{j}$ is a Wiener process]. Finally, there are two types of friction force: one between the monolayer and the substrate, characterized by

[6] $$\begin{array}{c} f_{i}^{\text{sub}}=-\Gamma \int d^{2}r\;\phi_{i}V\left(r\right)\;, \end{array}$$

with $V\left(r\right)=\Phi^{-1}\sum_{j=1}^{N}\phi_{j}v_{j}$ the tissue velocity field (48), and another one between the cells, expressed as

[7] $$\begin{array}{c} f_{i}^{\text{vis}}=\eta \sum_{j=1}^{N}\int d^{2}r\;I\left(\phi_{i},\phi_{j}\right)\cdot \left[V\left(r\right)-v_{j}\right]\;, \end{array}$$

where

[8] $$\begin{array}{c} I_{\alpha \beta }\left(\phi_{i},\phi_{j}\right)=\frac{1}{\Phi }\left[\left(\partial_{\gamma }\phi_{i}\right)\left(\partial_{\gamma }\phi_{j}\right)\delta_{\alpha \beta }+\left(\partial_{\alpha }\phi_{i}\right)\left(\partial_{\beta }\phi_{j}\right)\right] \end{array}$$

is a tensor related to the degree of interfacial overlap between cells i and j (i.e., cells only experience friction when they are close to each other). This expression for the cell–cell friction can be derived by considering the tissue as a viscous, compressible medium that can swell with fluid intake (hence the appearance of both bulk and shear terms in I) (48). One can show that, in the limit where three-field overlaps are rare, this form of friction can be approximated as a sum of pairwise friction proportional to $v_{i}-v_{j}$. To gain some intuition of this friction force, in *SI Appendix*, Fig. S1 we show how varying it affects $v_{i}$ during the head-on collision between two cells. A full list of the parameter values used in this work and the numerical procedure for solving for $v_{i}$ and $\phi_{i}$ are provided in *SI Appendix*.

## Supplementary Material

Appendix 01 (PDF)

Movie S1. Time evolution of the number of nearest neighbors *N*_nn_ of each cell within a monolayer of *N* = 400 cells for $\left(\xi_{n}/R,\xi_{p}/R\right)$ = (1.4, 0.2).

Movie S2. Time evolution of the number of nearest neighbors *N*_nn_ of each cell within a monolayer of *N* = 400 cells for $\left(\xi_{n}/R,\xi_{p}/R\right)$ = (1.0, 0.6).

Movie S3. Time evolution of the number of nearest neighbors *N*_nn_ of each cell within a monolayer of *N* = 400 cells for $\left(\xi_{n}/R,\xi_{p}/R\right)$ = (0.6, 1.0).

Movie S4. Time evolution of the angle *θ* between the deformation axis and the *x*-axis of each cell within a monolayer of *N* = 400 cells for $\left(\xi_{n}/R,\xi_{p}/R\right)$ = (1.4, 0.2).

Movie S5. Time evolution of the angle *θ* between the deformation axis and the *x*-axis of each cell within a monolayer of *N* = 400 cells for $\left(\xi_{n}/R,\xi_{p}/R\right)$ = (1.0, 0.6).

Movie S6. Time evolution of the angle *θ* between the deformation axis and the x-axis of each cell within a monolayer of *N* = 400 cells for $\left(\xi_{n}/R,\xi_{p}/R\right)$ = (0.6, 1.0).

Movie S7. Time evolution of the director field derived from the ***Q*** tensor, the nematic ±1/2 defects (+1/2 marked by red tadpoles and −1/2 by blue three-edge stars), and the hexatic 5–7 disclinations (cells with fivefold disclination in light blue and those with sevenfold in orange) for $\left(\xi_{n}/R,\xi_{p}/R\right)$ = (1.0, 0.6), where we find large local nematic ordering. The simulated monolayer has *N* = 400 cells.

## Data Availability

Simulation code and data for the figures have been deposited in Edinburgh DataShare (https://doi.org/10.7488/ds/7799) (49).
