# Supplementary material for: Intercellular friction and motility drive orientational order in cell monolayers
Source: Proc Natl Acad Sci U S A. 2024 Sep 20;121(40):e2319310121. doi: 10.1073/pnas.2319310121 (PMC11459176; doi:10.1073/pnas.2319310121)
Supplement: Supplementary file 1 — Appendix 01 (PDF) [file pnas.2319310121.sapp.pdf]

Supporting Information for

Intercellular friction and motility drive orientational order in cell monolayers

Michael Chiang, Austin Hopkins, Benjamin Loewe, M. Cristina Marchetti, Davide Marenduzzo

Michael Chiang, Austin Hopkins.
E-mail: michael.chiang@ed.ac.uk, austinhopkins@ucsb.edu

This PDF file includes:

Supporting text
Figs. S1 to S12
Table S1
Legends for Movies S1 to S7
SI References

Other supporting materials for this manuscript include the following:

Movies S1 to S7

Supporting Information Text

Methods

In the following, we first discuss the derivation of the chemical potential and the advection velocity of each cell, as they are involved in the equations of motion of the phase fields. We then provide additional information on the simulation procedure, as well as the full list of model parameters and the values used. Finally, we explain more in detail the definition and computation of some of the structural and dynamical observables measured in the simulations.

Computing the chemical potential μ_i. Within our monolayer model, it is necessary to explicitly compute the chemical potential of a cell $\mu_i = \delta\mathcal{F}/\delta\phi_i$, as it is required for updating the passive force $\boldsymbol{f}_i^{\text{pas}}$ acting upon the cell and for evolving the phase field ϕ_i over time. Recall that the free energy of the system is given by

$$\mathcal{F} = \sum_{i=1}^{N} \left\{ \int d^2\boldsymbol{r}\, \kappa \left[\phi_i^2(\phi_i - 1)^2 + \xi^2(\boldsymbol{\nabla}\phi_i)^2\right] + \lambda A_0\, \delta A_i^2[\phi_i] + \epsilon \sum_{i<j=1}^{N} \int d^2\boldsymbol{r}\, \phi_i^2\phi_j^2 \right\} , \quad \text{[S1]}$$

with $A_0 = \pi R^2$ and

$$\delta A_i[\phi_i] = 1 - \int d^2\boldsymbol{r}\, \frac{\phi_i^2}{A_0} . \quad \text{[S2]}$$

Taking the functional derivative with respect to ϕ_i, we find

$$\mu_i = \frac{\delta\mathcal{F}}{\delta\phi_i} = 2\kappa\left[\phi_i(\phi_i - 1)(2\phi_i - 1) - \xi^2(\nabla^2\phi_i)\right] - 4\lambda\phi_i\delta A_i + 2\epsilon\phi_i\left[h(\boldsymbol{r}) - \phi_i^2\right] , \quad \text{[S3]}$$

where $h(\boldsymbol{r}) = \sum_{j=1}^{N}\phi_j^2(\boldsymbol{r})$ is an auxiliary field introduced to enable parallel computation of individual phase fields.

Solving for the cell advection velocity $\boldsymbol{v}_i$. As discussed in the main text, we impose force balance within the model [Eq. (3)], which can be written as

$$-f_{i,\alpha}^{\text{sub}}(\{\phi_j\},\{\boldsymbol{v}_j\}) - f_{i,\alpha}^{\text{vis}}(\{\phi_j\},\{\boldsymbol{v}_j\}) = f_{i,\alpha}^{\text{pas}}(\{\phi_j\}) + f_{i,\alpha}^{\text{pol}}(\{\phi_j\}) . \quad \text{[S4]}$$

Here, we use Einstein summation for the Greek indices denoting Cartesian components, whereas there is no implicit summation for the Latin cell indices. To solve for the advection velocity, we write all velocity-dependent forces explicitly in terms of $v_{i,\alpha}$. The challenge in doing so lies in the intercellular friction term, which contains the total tissue velocity field V_α, i.e.,

$$f_{i,\alpha}^{\text{vis}} = -\eta\sum_{j=1}^{N}\int d^2\boldsymbol{r}\,\frac{1}{\Phi}\left[(\partial_\gamma\phi_i)(\partial_\gamma\phi_j)\delta_{\alpha\beta} + (\partial_\alpha\phi_i)(\partial_\beta\phi_j)\right](v_{j,\beta} - V_\beta) . \quad \text{[S5]}$$

To make progress, we observe that

$$\sum_{j=1}^{N}(\partial_\beta\phi_j)\,V_\beta = \sum_{j=1}^{N}(\partial_\beta\phi_j)\sum_{k=1}^{N}\frac{\phi_k}{\Phi}v_{k,\beta} = \sum_{j=1}^{N}\frac{\phi_j}{\Phi}(\partial_\beta\Phi)\,v_{j,\beta} , \quad \text{[S6]}$$

where we have swapped the summing indices in the second equality. Hence, one can write

$$\sum_{j=1}^{N}\int d^2\boldsymbol{r}\,\frac{\partial_\alpha\phi_i}{\Phi}(\partial_\beta\phi_j)(v_{j,\beta} - V_\beta) = \sum_{j=1}^{N}\int d^2\boldsymbol{r}\,\frac{\partial_\alpha\phi_i}{\Phi}\left[(\partial_\beta\phi_j) - \frac{\phi_j(\partial_\beta\Phi)}{\Phi}\right]v_{j,\beta} \equiv \sum_{j=1}^{N}K_{ij,\alpha\beta}\,v_{j,\beta} , \quad \text{[S7]}$$

and, by the same procedure,

$$\sum_{j=1}^{N}\int d^2\boldsymbol{r}\,\frac{\partial_\gamma\phi_i}{\Phi}(\partial_\gamma\phi_j)\delta_{\alpha\beta}(v_{j,\beta} - V_\beta) = \sum_{j=1}^{N}K_{ij,\gamma\gamma}\delta_{\alpha\beta}\,v_{j,\beta} , \quad \text{[S8]}$$

such that

$$f_{i,\alpha}^{\text{vis}} = -\eta\sum_{j=1}^{N}(K_{ij,\gamma\gamma}\delta_{\alpha\beta} + K_{ij,\alpha\beta})\,v_{j,\beta} . \quad \text{[S9]}$$

Noting that the cell–substrate friction term can be written as

$$f_{i,\alpha}^{\text{sub}} = -\Gamma\sum_{j=1}^{N}\int d^2\boldsymbol{r}\,\frac{\phi_i\phi_j}{\Phi}\,v_{j,\alpha} = -\Gamma\sum_{j=1}^{N}\hat{O}_{ij}\delta_{\alpha\beta}\,v_{j,\beta} , \quad \text{[S10]}$$

with

$$\hat{O}_{ij} \equiv \int d^2\boldsymbol{r}\, \frac{\phi_i \phi_j}{\Phi}\,, \tag{S11}$$

the force balance equation can therefore be expressed as

$$\sum_{j=1}^{N} \left[(\eta K_{ij,\gamma\gamma} + \Gamma \hat{O}_{ij}) \delta_{\alpha\beta} + \eta K_{ij,\alpha\beta} \right] v_{j,\beta} = f_{i,\alpha}^{\text{pas}} + f_{i,\alpha}^{\text{pol}} \equiv u_{i,\alpha}\,, \tag{S12}$$

where

$$u_{i,\alpha} = -\sum_{j=1}^{N} \int d^2\boldsymbol{r}\, \phi_i \phi_j (\partial_\alpha \mu_j) + \Gamma v_0 \sum_{j=1}^{N} \hat{O}_{ij}\, p_{j,\alpha}\,. \tag{S13}$$

This is a set of linear equations in $v_{j,\beta}$ that can be cast in a matrix form. Specifically, by defining

$$\boldsymbol{A}_{ij} \equiv \left[\eta\, \mathrm{Tr}(\boldsymbol{K}_{ij}) + \Gamma \hat{O}_{ij} \right] \boldsymbol{I} + \eta\, \boldsymbol{K}_{ij} \tag{S14}$$

and writing $\boldsymbol{v}_i = (v_{i,x}, v_{i,y})^T$ and $\boldsymbol{u}_i = (u_{i,x}, u_{i,y})^T$, Eq. (S12) can be written as

$$\begin{pmatrix} \boldsymbol{A}_{11} & \boldsymbol{A}_{12} & \cdots & \boldsymbol{A}_{1N} \\ \boldsymbol{A}_{21} & \boldsymbol{A}_{22} & \cdots & \boldsymbol{A}_{2N} \\ \vdots & \vdots & \ddots & \vdots \\ \boldsymbol{A}_{N1} & \boldsymbol{A}_{N2} & \cdots & \boldsymbol{A}_{NN} \end{pmatrix} \begin{pmatrix} \boldsymbol{v}_1 \\ \boldsymbol{v}_2 \\ \vdots \\ \boldsymbol{v}_N \end{pmatrix} = \begin{pmatrix} \boldsymbol{u}_1 \\ \boldsymbol{u}_2 \\ \vdots \\ \boldsymbol{u}_N \end{pmatrix}, \tag{S15}$$

which can be inverted numerically to solve for $v_{i,\alpha}$.

Additional details of the simulation procedure. We consider different initial conditions and system sizes for the simulations. For $N = 100$ cells, we examine both the case where cells are randomly initialized (with box size $L_x = L_y = 145$) and the case where they are initially positioned on a triangular lattice (spacing $2R$, and the box dimensions are $L_x = 156$ and $L_y = 135$; this aspect ratio preserves the regularity of the lattice). For $N = 400$, we only perform simulations for a random initial configuration (with size $L_x = L_y = 290$; see *SI Appendix*, Fig. S2 for snapshots of these different initial conditions and system sizes). The box dimensions are chosen to give a pack fraction of approximately 0.95. Unless otherwise stated, the results presented in the main text are for the case of $N = 100$ cells that are initialized randomly, whereas the results for other conditions are shown here in *SI Appendix*.

We solve the matrix equations for $\boldsymbol{v}_i$ [Eq. (S15)] using standard `LAPACK` routines, and we numerically integrate the equations of motion for ϕ_i [Eq. (2)] using a third-order upwind finite difference scheme. To increase computational efficiency, we employ the domain decomposition strategy when updating individual phase fields (the size of the subdomain is $L_x^{\text{sub}} = L_y^{\text{sub}} = 35$), and the simulations are parallelized using `OpenMP`. The full details of this parallelization strategy have been discussed in our previous work (1).

In each simulation, the system is first allowed to relax and equilibrate for 10^5 timesteps, during which cell motility and friction forces are switched off. Afterwards, all forces are turned on, and the simulation runs for 10^6 timesteps for further equilibration. Finally, the simulation is run for 10^7 timesteps with the structural and dynamical properties of the monolayer sampled every 10^3 timesteps. We perform multiple runs (10 for $N = 100$ and 5 for $N = 400$) for each parameter point of the phase diagram. Unless otherwise stated, ensemble averages $\langle \cdot \rangle$ of observables are computed by averaging over both time and simulation runs.

Simulation parameters and their mappings to physical values. In *SI Appendix*, Table S1, we list the full set of model parameters and their values in the simulations. To map the parameter values from simulation to physical units, one needs to fix the simulation length δx, time δt, and energy scale δE based on some fundamental quantities that characterize a cell monolayer, which we take to be the radius R of a cell, its persistence time D_r^{-1}, and its edge tension σ. We estimate the physical values of these quantities using data on mammary epithelial MCF-10A cells (other cell types will give similar values), and we find $R \sim 10$ μm and $D_r^{-1} \sim 1$ hr based on cell and particle image velocimetry experiments (2). Taking the cortical tension of MCF-10A cells to be ~0.4 mN/m, as found from oscillatory atomic force microscopy (3), and the typical cell height as 6 μm, we estimate the edge tension to be $\sigma \sim 0.4$ mN/m $\times$ 6 μm = 2.4 nN. To resolve the contours of individual cells and ensure numerical stability when integrating the equations of motion, we choose $R = 8\,\delta x$, $D_r^{-1} = 1000\,\delta t$, and $\sigma = 0.002\,\delta E/\delta x$ in our simulations. Using the estimates above thus gives $\delta x = 1.25$ μm, $\delta t = 3.6$ s, and $\delta E = 1.5$ mm·nN. Values of other parameters are mapped from simulation to physical units based on these scales. To check the validity of our parameters, we use Γv_0 as a typical scale of cell traction forces and find ~10–130 Pa, which is reasonable for epithelial cells on a substrate.

Parameter	Interpretation	Dimensions	Value(s) in simulation units	Value(s) in physical units
δx	Size of each lattice pixel	$[L]$	1	1.25 μm
δt	Size of each timestep	$[T]$	1	3.6 s
δE	Energy scale	$[E]$	1	1.5 mm·nN
σ	Cell-edge tension	$[E][L]^{-1}$	0.002	2.4 nN
ξ	Interfacial thickness	$[L]$	1	1.25 μm
λ	Area constraint	$[E][L]^{-2}$	0.1	96 nN/μm
ϵ	Cell–cell repulsion	$[E][L]^{-2}$	0.1	96 nN/μm
R	Ideal cell radius	$[L]$	8	10 μm
D_r	Rotational diffusion rate	$[T]^{-1}$	0.001	1 hr^{-1}
Γ	Cell–substrate friction	$[E][T][L]^{-4}$	0.0156	34.5 kPa·s/μm
η	Cell–cell friction	$[E][T][L]^{-2}$	0.0–4.0	0–13.8 MPa·s·μm
v_0	Cell motility	$[L][T]^{-1}$	0.0008–0.0112	1–14 μm/hr

Table S1. A full list of the model parameters, their dimensions, and their values both in simulation and physical units. Note that in simulation units, length is expressed in units of the grid-point spacing δx, time in units of the simulation timestep δt, and energy in units of δE.

Characterizing the monolayer's structure and dynamics in the solid–liquid transition. When analyzing the solid–liquid transition, we classify the monolayer to be in the solid or liquid phase based on the average effective diffusivity D_{eff} of the cells. In practice, this is computed by performing linear fits to the mean square displacement curves at large lag times ($D_r t = 4000$ to 8000) and using the slope of the fit to estimate the diffusivity. We then use the threshold $D^*_{\text{eff}} = 10^{-3}$ to distinguish between the solid and the liquid phase, as this matches well with results from structural observables.

As part of the analysis of the monolayer's structure, we compute the global translational order parameter Ψ_T, in addition to the global bond-orientational (hexatic) order parameter Ψ_6. This parameter is defined as

$$\Psi_T = \left\langle \left| \frac{1}{N} \sum_{j=1}^{N} \exp\left(i\boldsymbol{q}\cdot\boldsymbol{r}_j\right) \right| \right\rangle , \qquad \text{[S16]}$$

where $\boldsymbol{r}_j$ is the center of mass of cell j and $\boldsymbol{q}$ is a reciprocal lattice vector. The ensemble average here is computed over the reciprocal lattice vectors for a regular triangular lattice, i.e., $\boldsymbol{q}_1 = \frac{\pi}{R}\left(1, \frac{1}{\sqrt{3}}\right)$ and $\boldsymbol{q}_2 = \frac{\pi}{R}\left(0, \frac{2}{\sqrt{3}}\right)$, as well as over time and simulation runs. Note that $\Psi_T \sim 1$ when the system exhibits crystalline ordering, and this is only found in our simulations when cells within the monolayer are initialized on a lattice (*SI Appendix*, Figs. S3, S6*C*, and S7*C*).

To quantify glassy dynamics, we compute two observables. One of them is the self-intermediate scattering function $F_s(\boldsymbol{q}, t)$, which monitors the relaxation of the system's structural ordering and is written as

$$F_s(\boldsymbol{q}, t) = \left\langle \left| \frac{1}{N} \sum_{j=1}^{N} \exp\left[i\boldsymbol{q}\cdot[\boldsymbol{r}_j(t) - \boldsymbol{r}_j(0)]\right] \right| \right\rangle , \qquad \text{[S17]}$$

where the average is taken over multiple wavevectors $\boldsymbol{q}$ with the same magnitude $|\boldsymbol{q}| = \pi/R$ and over lag time and simulation runs. Systems with glassy dynamics typically exhibit two relaxation regimes, as observed in our simulations (Fig. 2*B* and *SI Appendix*, Figs. S4*A* and S5). The other observable is the non-Gaussian parameter α_2 and is given by

$$\alpha_2 = \frac{1}{2} \frac{\left\langle \frac{1}{N}\sum_{i=1}^{N} \Delta\boldsymbol{r}_i^4 \right\rangle}{\left\langle \frac{1}{N}\sum_{i=1}^{N} \Delta\boldsymbol{r}_i^2 \right\rangle^2} - 1 , \qquad \text{[S18]}$$

where $\Delta\boldsymbol{r}_i = \boldsymbol{r}_i(t) - \boldsymbol{r}_i(0)$ and the average is taken over lag time and simulation runs. This typically peaks in the parameter regime where cells are experiencing transient caging effects (*SI Appendix*, Fig. S4*B*).

Quantifying the size of local nematic domains in cell deformation and cellular flow. To estimate the size of local domains where we find nematic alignment in the cell, we first define a set of rectangular grid points G within the monolayer (10×10 points, equally spaced; note that these points generally do not coincide with cell positions). For each grid point p, we then compute the degree of nematic ordering of the deformation axes Ψ_d and of the advection velocities Ψ_v of all the cells within a threshold radius r from the point (we use the distance between each cell's center of mass and the grid point; *SI Appendix*, Fig. S9*A*), and we average this result across all grid points. Mathematically, this local order can be expressed as

$$\Psi_{d,v}(r) = \left\langle \frac{1}{N_p} \sum_{p\in G} \left| \frac{1}{N_r^p} \sum_{j=1}^{N} \Theta(r - |\boldsymbol{r}_j - \boldsymbol{r}_p|) \exp\left(i2\theta_j^{d,v}\right) \right| \right\rangle , \qquad \text{[S19]}$$

where $\theta_j^{d,v}$ is the angle that the deformation axis or the advection velocity of cell j makes with the x-axis, $\boldsymbol{r}_p$ is the position of the grid point p, N_p is the number of grid points, $N_r^p = \sum_{k=1}^{N} \Theta(r - |\boldsymbol{r}_k - \boldsymbol{r}_p|)$, and $\Theta(x)$ is the Heaviside step function. To determine the domain size, we vary r to obtain a decay curve of $\Psi_{d,v}$ as a function of this threshold (*SI Appendix*, Fig. S9*B* and *D*). Finally, we define a threshold in the ordering (based on the maximum global order observed when considering the entire system) to obtain a characteristic length $\xi_{d,v}$ (*SI Appendix*, Fig. S9*C* and *E*).

$\boldsymbol{Q}$ tensor definition, the director field, and nematic defects identification. As mentioned in the results section, the $\boldsymbol{Q}$ tensor is defined as

$$Q_{\alpha\beta}(\boldsymbol{r}) = \sum_{i=1}^{N} \mathcal{W}_i(\boldsymbol{r})\, \mathcal{S}_i (2n_{i,\alpha} n_{i,\beta} - \delta_{\alpha\beta}) \,. \tag{S20}$$

Here, $\mathcal{S}_i$ is the degree of deformation of cell i and is given by

$$\mathcal{S}_i = \frac{\lambda_{i,+} - \lambda_{i,-}}{\lambda_{i,+} + \lambda_{i,-}} \,, \tag{S21}$$

where $\lambda_{i,\pm}$ are the eigenvalues of the shape tensor $\boldsymbol{S}_i$, and $\boldsymbol{n}_i$ is the eigenvector associated with $\lambda_{i,+}$ (the deformation axis). $\mathcal{W}_i(\boldsymbol{r})$ is an ellipsoidal smoothing function defined as

$$\mathcal{W}_i(\boldsymbol{r}) = \frac{1}{2}\left[1 + \tanh\left[1 - \left(\frac{x'}{\lambda_{i,+}}\right)^2 - \left(\frac{y'}{\lambda_{i,-}}\right)^2\right]\right] , \tag{S22}$$

where x' and y' are Cartesian coordinates in the frame where the x-axis aligns with the deformation axis of the cell and the origin coincides with the cell's center of mass. When plotting the director field and identifying nematic defects, we further smooth the $\boldsymbol{Q}$ field using a $3R \times 3R$ rolling window—i.e., we center the window at each grid point and replace $Q_{\alpha\beta}$ at that point by the average over all points within the window. To locate defects, we first mark the positions of the local minima in the eigenvalue field derived from this smoothed $\boldsymbol{Q}$ as candidate defects, since these will be the locations where the tissue is close to an isotropic state. Next, the topological charge is computed around each candidate defect using a square contour of width $3\,\delta x$, and only those that give a charge consistent with that of a nematic defect are retained. To reduce spurious detection, candidates (with the same topological charge) that are closer than a threshold of $2.5\,\delta x$ are clustered and treated as a single defect. This defect identification algorithm is highly robust, as the resulting set of defects obeys the conservation of topological charge (which is zero for a 2D periodic planar surface).

Supplemental Figures

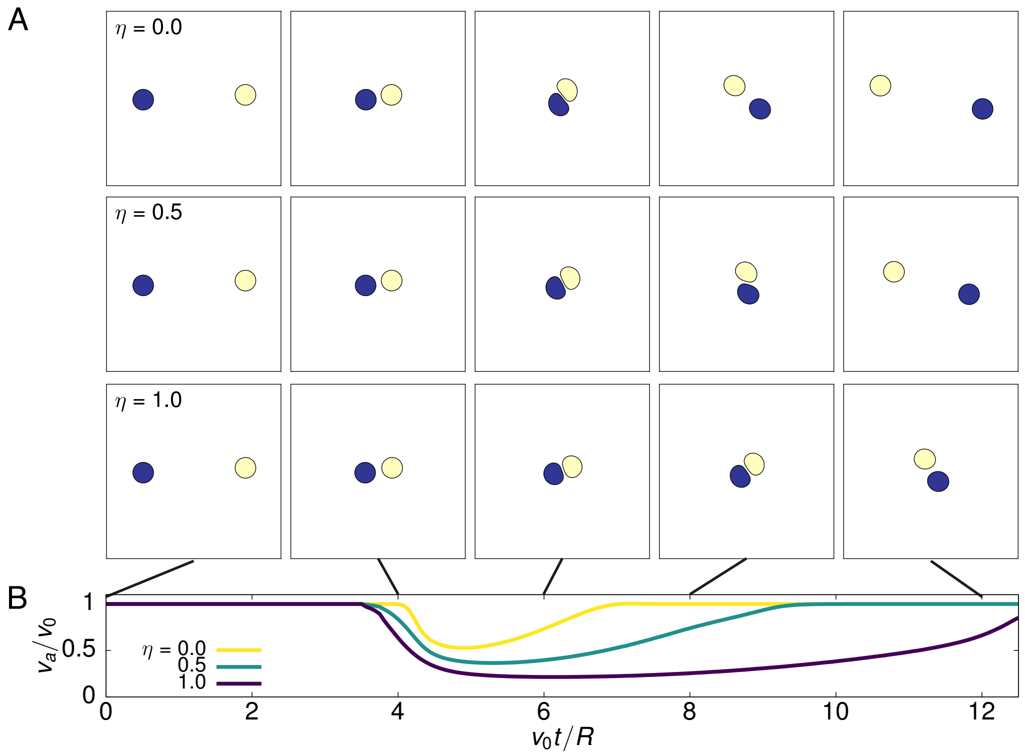

Fig. S1. Two-body collision simulations highlighting the effect of cell–cell friction (strength η). (*A*). Simulation snapshots at several time points during the collision process, for three η values. Here, the two cells move towards each other with a self-propulsion force that is proportional to $v_0 = 0.005$. The cells are initialized with their center of mass slightly off-centered in the y direction (by two grid points), so that they can still move past each other when colliding. (*B*) The magnitude of the advection velocity v_a, normalized by v_0, of the cells during the collision (a similar result is observed for the center-of-mass velocity). Note that the cells slow down more and the collision duration increases as the friction strength η becomes stronger.

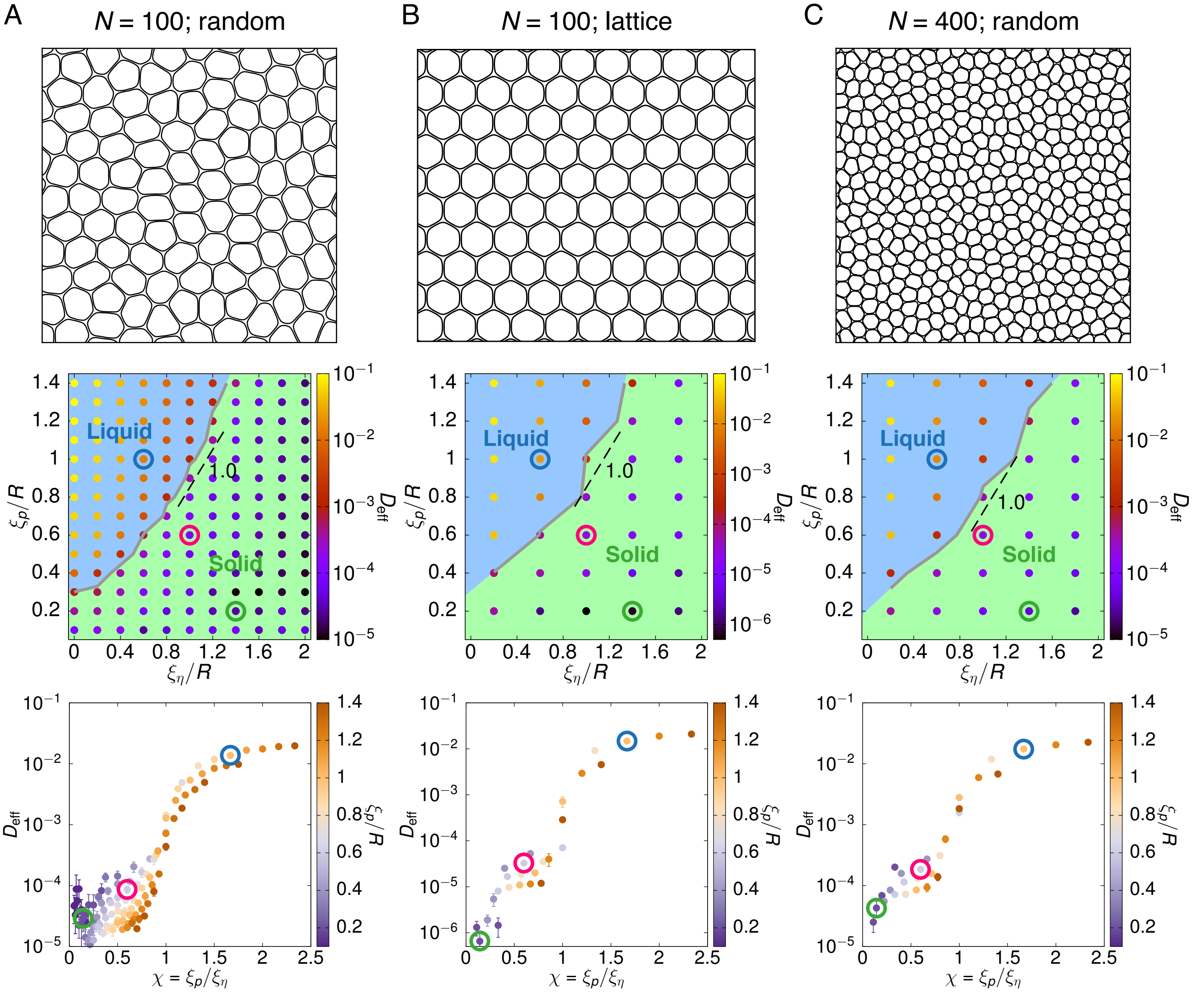

Fig. S2. Comparing the solid–liquid phase diagram for three different system setups. (*A*) $N = 100$ cells that are initialized randomly. (*B*) $N = 100$ cells that are initialized on a triangular lattice. (*C*) $N = 400$ cells that are initialized randomly. *Top*: The initial configuration of the cells. *Middle*: The phase diagram, with points colored by the measured effective diffusivity D_{eff}, and the transition boundary is based on the threshold $D_{\text{eff}} = 10^{-3}$. *Bottom*: Collapsing D_{eff} based on $\chi = \xi_p/\xi_\eta$ onto a master curve, for points where $\xi_\eta \geq 0.6$.

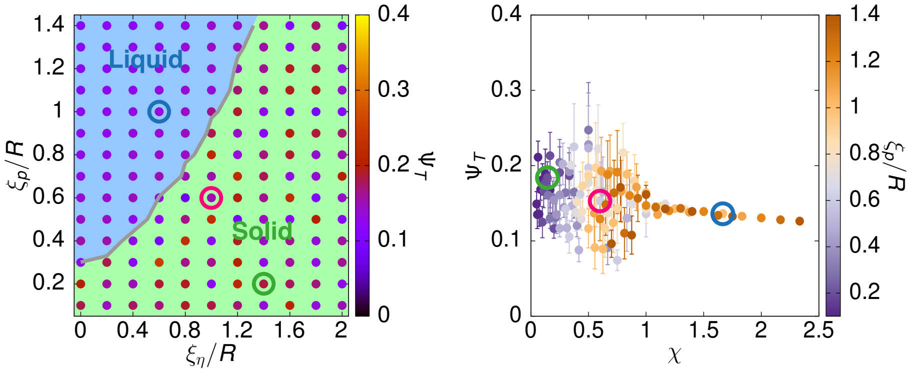

Fig. S3. Global translational order Ψ_T of the system ($N = 100$ cells, randomly initialized). *Left*: Measurements of Ψ_T superposed on the solid–liquid phase diagram shown in Fig. 1*B* (and *SI Appendix*, Fig. S2*A*). *Right*: Collapsing Ψ_T based on χ onto a master curve, for points where $\xi_\eta \geq 0.6$.

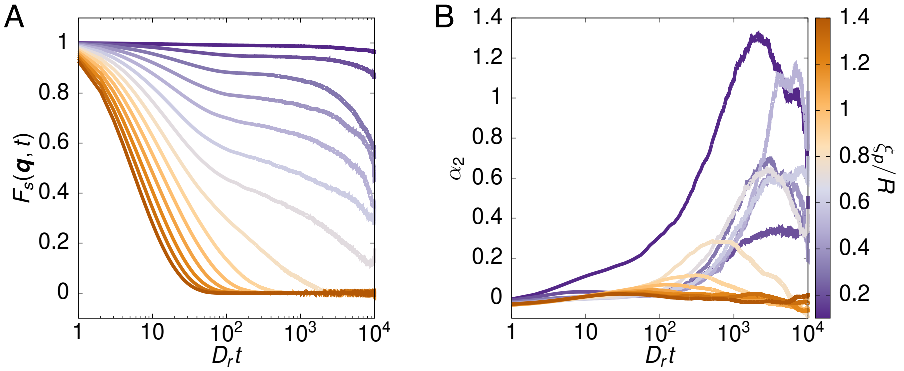

Fig. S4. Glassy dynamics in the solid regime of the monolayer. (*A*) Self-intermediate scattering function $F_s(\boldsymbol{q}, t)$ (with $|\boldsymbol{q}| = R$) and (*B*) non-Gaussian parameter α_2 as a function of lag time when varying χ (i.e., vary ξ_p/R at $\xi_\eta/R = 0.8$). Both plots are for the system size $N = 100$ cells.

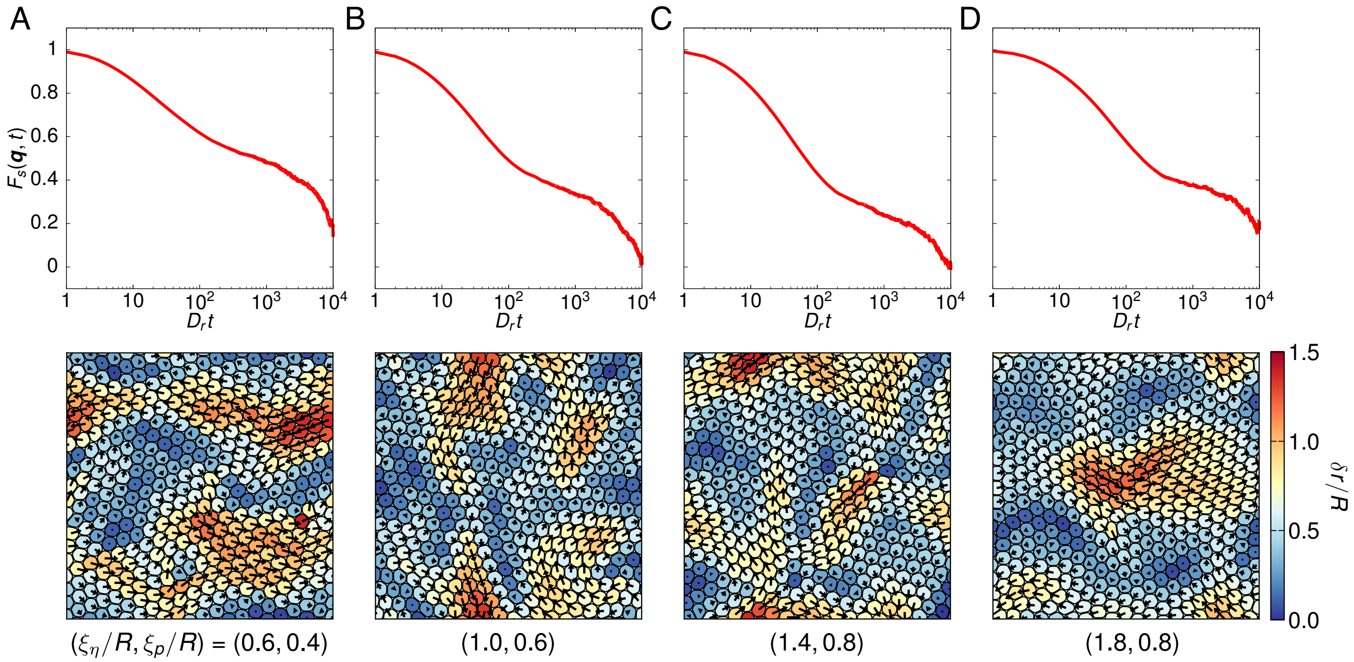

Fig. S5. Examples of local correlation in cell displacements in the glassy solid regime. Top panel shows the self-intermediate scattering function $F_s(\boldsymbol{q}, t)$ and the bottom panel displays a representative snapshot of cell displacements $\delta r/R$ between two time points where $F_s(\boldsymbol{q}, t)$ has decayed to $\sim 1/2$. Arrows indicate the directions of the cell displacements, with their length twice the magnitude of the actual displacements to aid visualization. The parameter points are (*A*) $(\xi_\eta/R, \xi_p/R) = (0.6, 0.4)$, (*B*) $(1.0, 0.6)$, (*C*) $(1.4, 0.8)$, and (*D*) $(1.8, 0.8)$, and the lag times used are $D_r t = 500$, 100, 50, and 200, respectively.

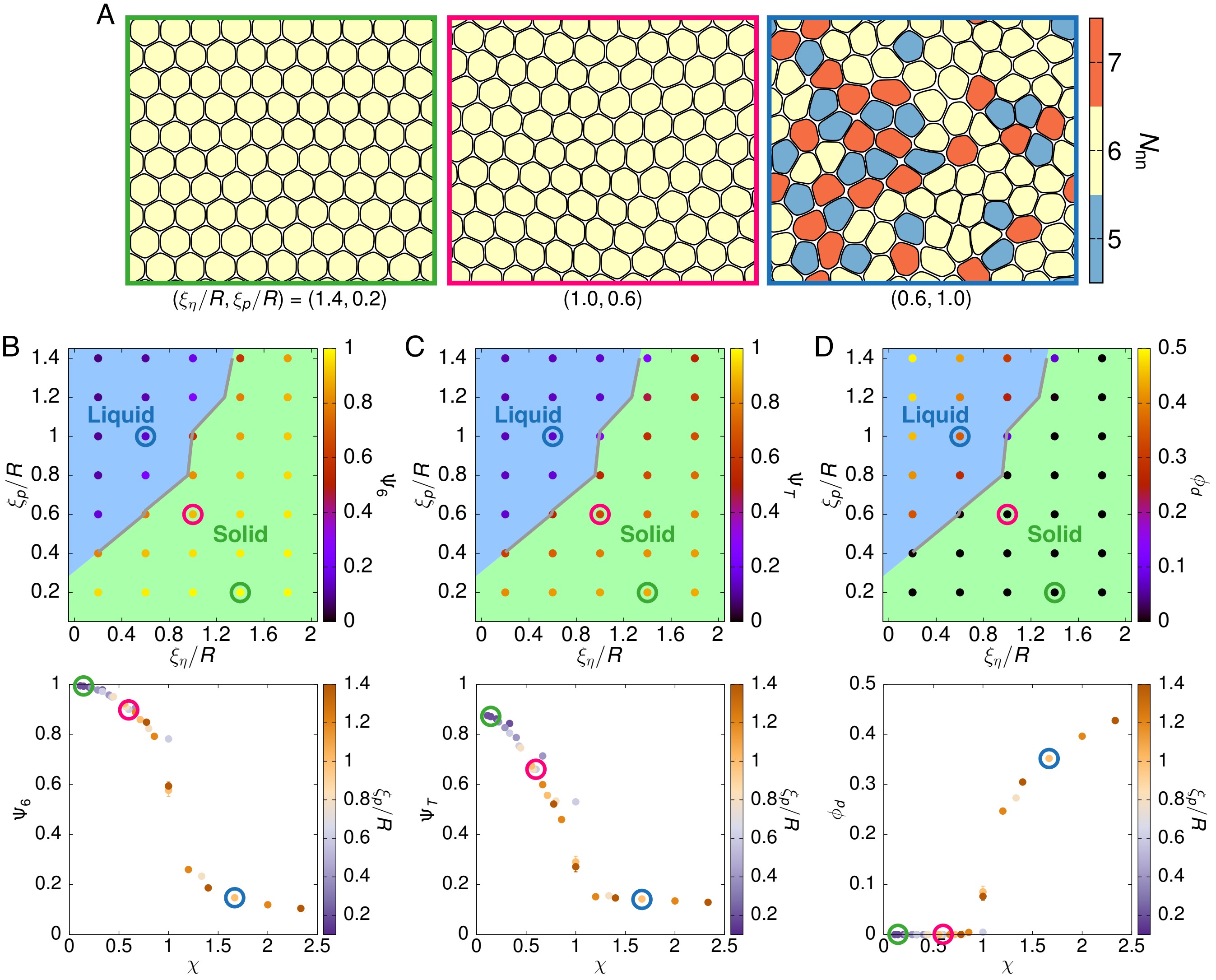

Fig. S6. Quantifying the degree of hexatic and translational order for the case of $N = 100$ cells that are initialized on a triangular lattice. (*A*) Simulation snapshots of three points in the phase space, with cells colored by their number of nearest neighbors N_{nn} (i.e., fivefold and sevenfold disclinations are marked in blue and orange, respectively). (*B–D*) *Top*: Measurements of the (*B*) global bond-orientational (hexatic) order parameter Ψ_6, (*C*) global translational order parameter Ψ_T, and (*D*) fraction of cells with disclinations ϕ_d superposed on the solid–liquid phase diagram shown in *SI Appendix*, Fig. S2*B*. *Bottom*: Collapsing these measurements based on χ onto a master curve, for points where $\xi_\eta \geq 0.6$.

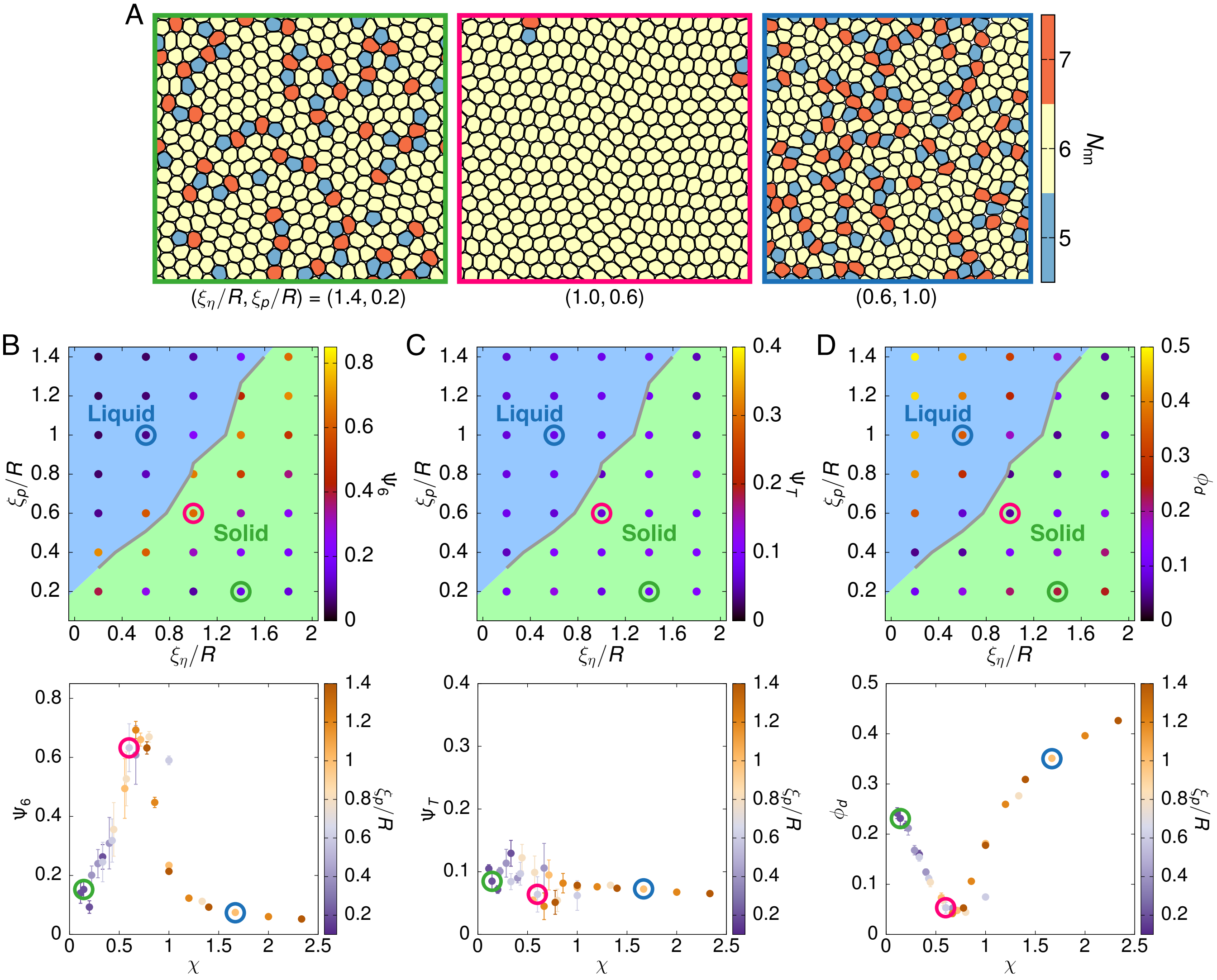

Fig. S7. Quantifying the degree of hexatic and translational order for the case of $N = 400$ cells that are initialized randomly. (*A*) Simulation snapshots of three points in the phase space, with cells colored by their number of nearest neighbors N_{nn} (i.e., fivefold and sevenfold disclinations are marked in blue and orange, respectively). (*B–D*) *Top*: Measurements of the (*B*) global bond-orientational (hexatic) order parameter Ψ_6, (*C*) global translational order parameter Ψ_T, and (*D*) fraction of cells with disclinations ϕ_d superposed on the solid–liquid phase diagram shown in *SI Appendix*, Fig. S2*C*. *Bottom*: Collapsing these measurements based on χ onto a master curve, for points where $\xi_\eta \geq 0.6$.

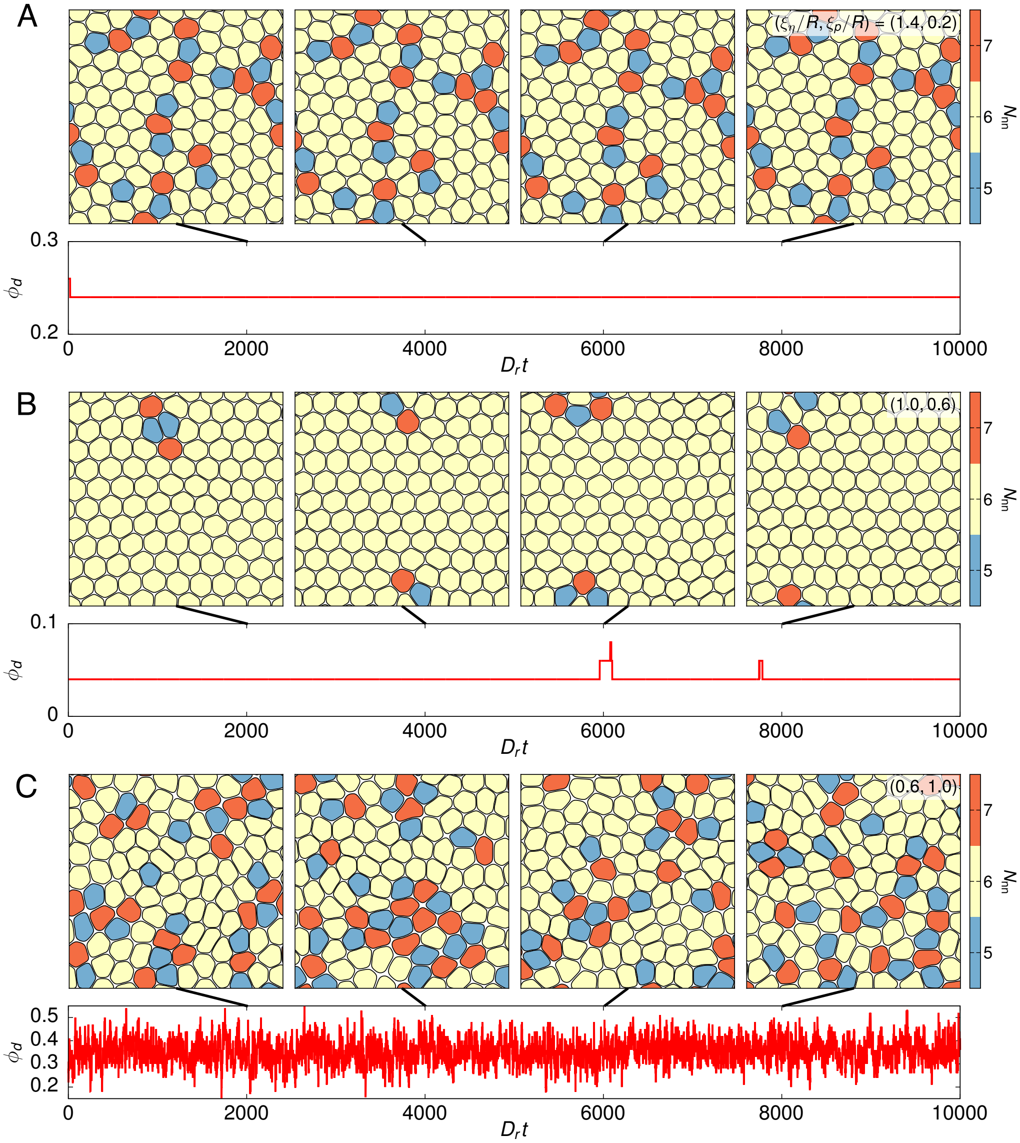

Fig. S8. Dynamics of 5–7 disclinations are distinct between liquid and solid phases. (*A*–*C*) Simulation snapshots showing the number of nearest neighbors N_{nn} for each cell at various time points (*Top*) and the time series reporting the fraction of cells with hexatic disclinations ϕ_d (*Bottom*) for three parameter points, corresponding to (*A*) the glassy solid regime, (*B*) the regime with high hexatic order, and (*C*) the liquid phase. See *SI Appendix*, Fig. S2*A* for the location of these points within the solid–liquid phase diagram. Data shown here are for the case of $N = 100$ cells that are initialized randomly.

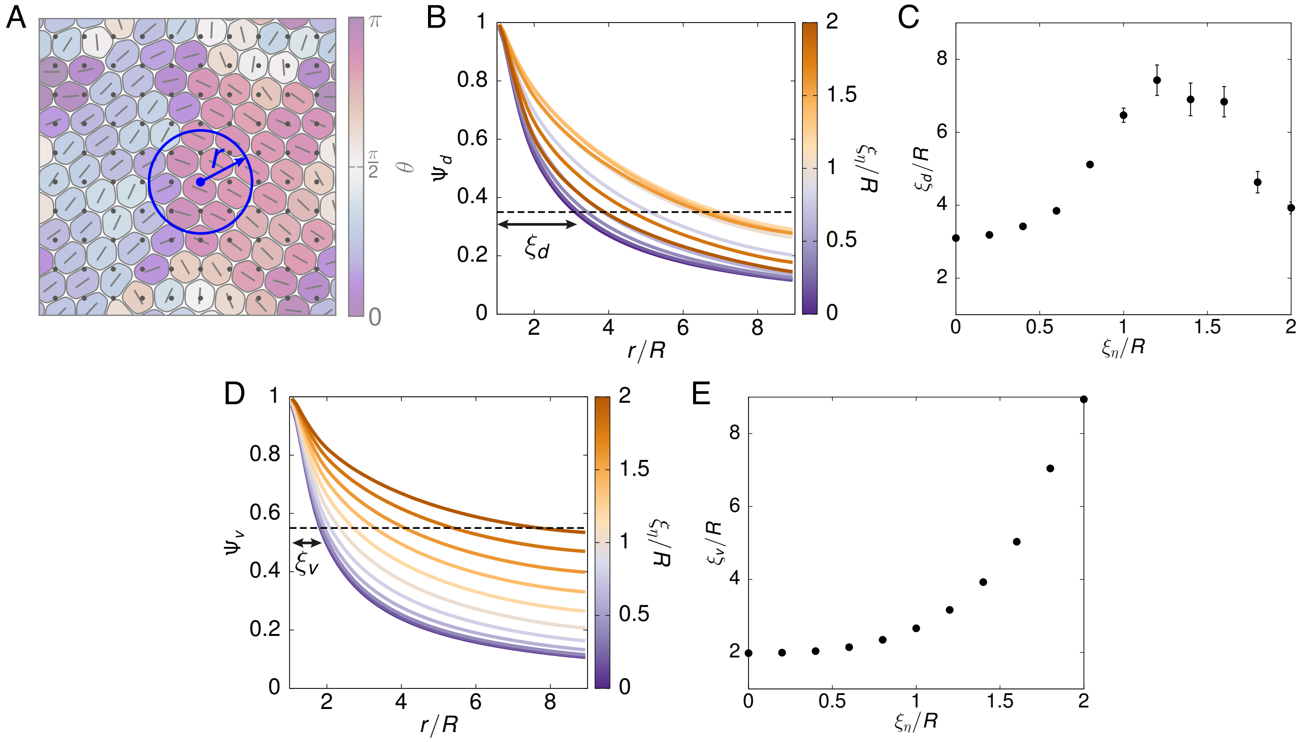

Fig. S9. Quantifying the extent of local nematic alignment in cell deformation and cellular flow. (*A*) Schematics explaining how the local nematic orders $\Psi_{d,v}$ are measured and used for extracting a characteristic length. Here, for each grid point (e.g., the one marked in blue), we compute the order parameter for cells within a radius r from the point and average the results over all grid points. (*B*) The local nematic order in cell deformation Ψ_d as a function of r. The curves are for different ξ_η, with fixed $\xi_p/R = 0.8$, and the dotted line marks the threshold $\Psi_d^* = 0.35$ used for extracting a characteristic length ξ_d. (*C*) The characteristic length ξ_d of the local nematic domain, as estimated from the curves shown in (*B*). (*D* and *E*) Similar to (*B* and *C*), but for the local nematic order in cell advection velocity Ψ_v (i.e., cellular flow). The threshold used for extracting a characteristic length ξ_v is $\Psi_v^* = 0.55$. Data shown here are for the case of $N = 100$ cells that are initialized randomly.

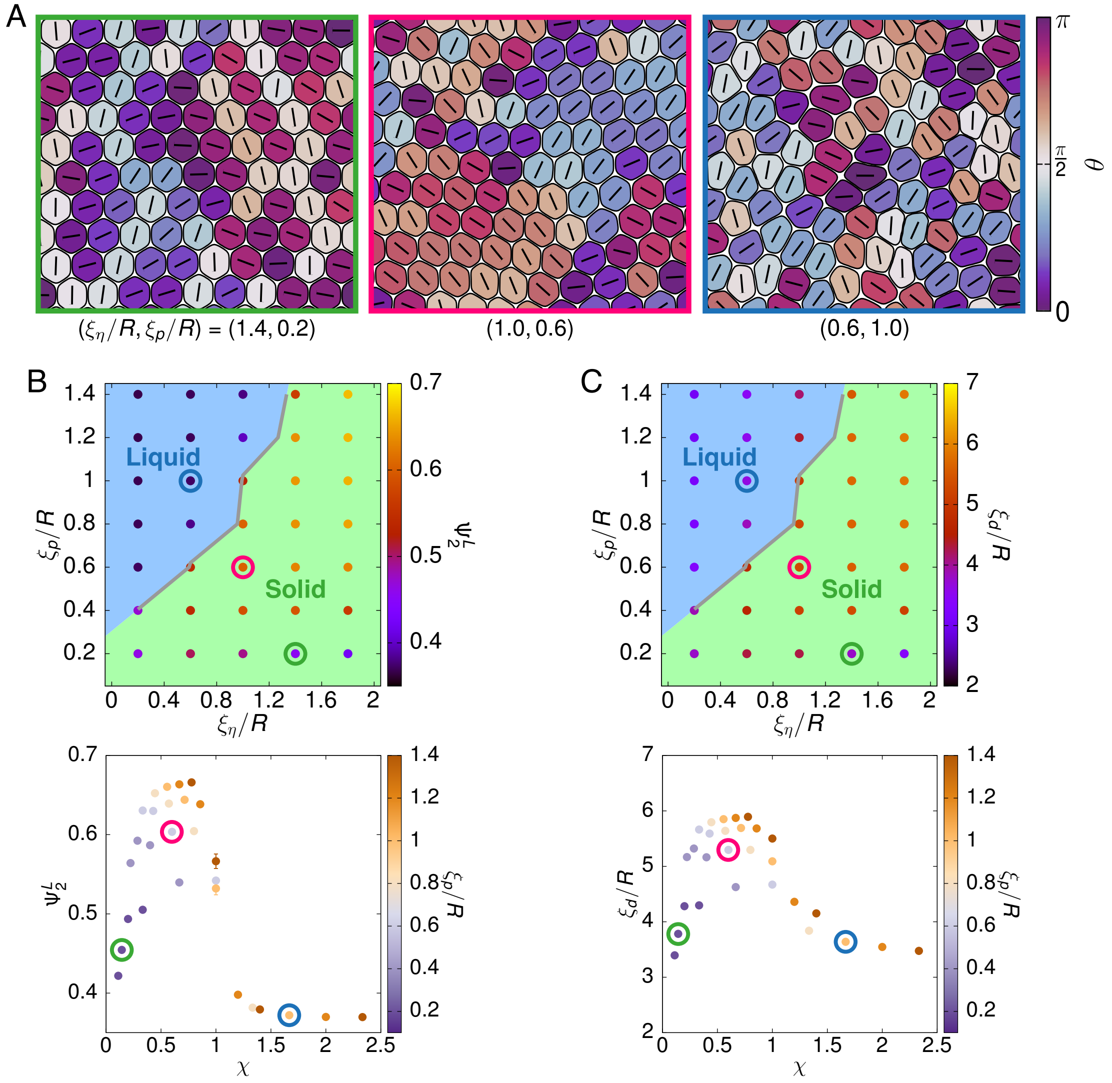

Fig. S10. Quantifying the degree of local nematic order in cell deformation for the case of $N = 100$ cells that are initialized on a triangular lattice. (*A*) Simulation snapshots of three points in the phase space, with cells colored by the angle θ between their deformation axis (the line within each cell) and the x-axis. (*B* and *C*) *Top*: Measurements of (*B*) the local nematic order parameter Ψ_2^L and (*C*) the characteristic length ξ_d of this local nematic order superposed on the solid–liquid phase diagram shown in *SI Appendix*, Fig. S2*B*. *Bottom*: Collapsing these measurements based on χ onto a master curve, for points where $\xi_\eta \geq 0.6$.

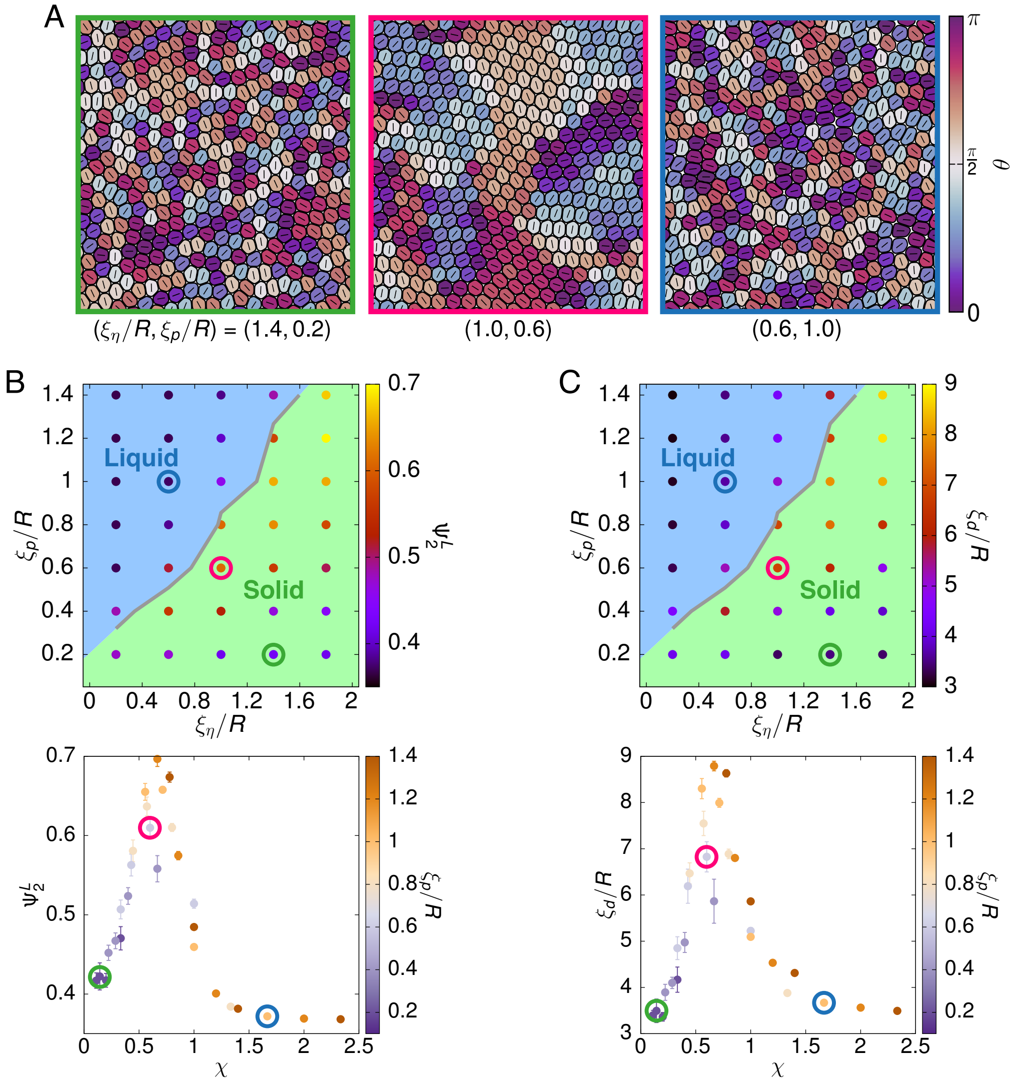

Fig. S11. Quantifying the degree of local nematic order in cell deformation for the case of $N = 400$ cells that are initialized randomly. (*A*) Simulation snapshots of three points in the phase space, with cells colored by the angle θ between their deformation axis (the line within each cell) and the x-axis. (*B* and *C*) *Top*: Measurements of (*B*) the local nematic order parameter Ψ_2^L and (*C*) the characteristic length ξ_d of this local nematic order superposed on the solid–liquid phase diagram shown in *SI Appendix*, Fig. S2*C*. *Bottom*: Collapsing these measurements based on χ onto a master curve, for points where $\xi_\eta \geq 0.6$.

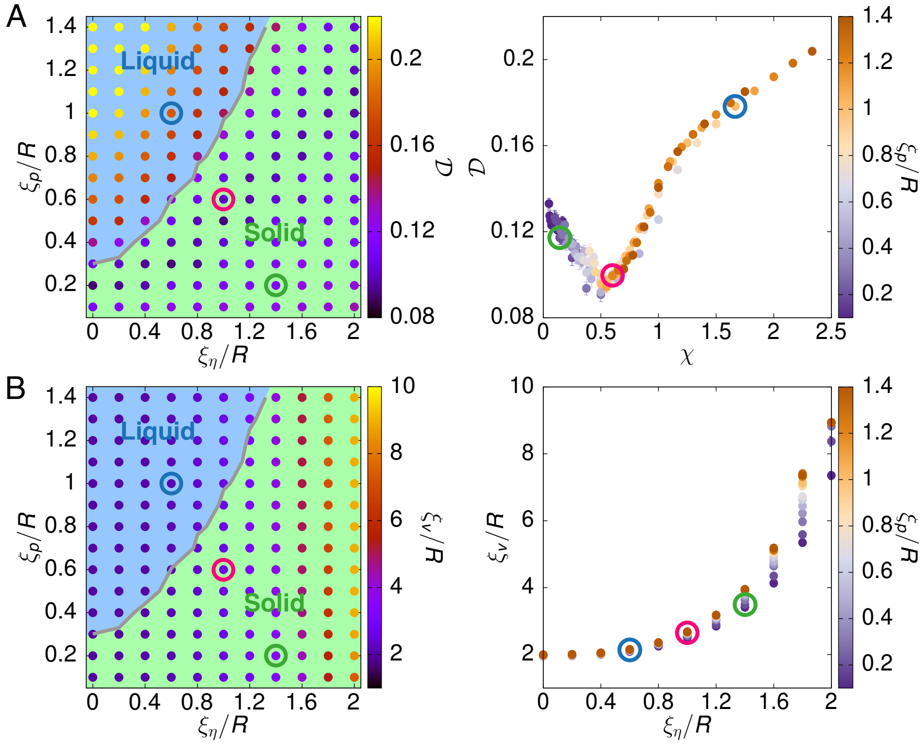

Fig. S12. The global degree of cell deformation $\mathcal{D}$ (*A*) and the characteristic length ξ_v of the local nematic order in cell advection velocity (*B*; see also *SI Appendix*, Fig. S9*D* and *E*) across the phase space. *Left*: Measurements of these observables superposed on the solid–liquid phase diagram shown in Fig. 1*B*. *Right*: Collapsing these measurements onto a master curve, based on χ, for points where $\xi_\eta \geq 0.6$, in (*A*) and based on ξ_η in (*B*). Data shown here are for the case of $N = 100$ cells that are initialized randomly.

Supplemental Movies

Movie S1. Time evolution of the number of nearest neighbors N_{nn} of each cell within a monolayer of $N = 400$ cells for $(\xi_\eta/R, \xi_p/R) = (1.4, 0.2)$.

Movie S2. Time evolution of the number of nearest neighbors N_{nn} of each cell within a monolayer of $N = 400$ cells for $(\xi_\eta/R, \xi_p/R) = (1.0, 0.6)$.

Movie S3. Time evolution of the number of nearest neighbors N_{nn} of each cell within a monolayer of $N = 400$ cells for $(\xi_\eta/R, \xi_p/R) = (0.6, 1.0)$.

Movie S4. Time evolution of the angle θ between the deformation axis and the x-axis of each cell within a monolayer of $N = 400$ cells for $(\xi_\eta/R, \xi_p/R) = (1.4, 0.2)$.

Movie S5. Time evolution of the angle θ between the deformation axis and the x-axis of each cell within a monolayer of $N = 400$ cells for $(\xi_\eta/R, \xi_p/R) = (1.0, 0.6)$.

Movie S6. Time evolution of the angle θ between the deformation axis and the x-axis of each cell within a monolayer of $N = 400$ cells for $(\xi_\eta/R, \xi_p/R) = (0.6, 1.0)$.

Movie S7. Time evolution of the director field derived from the $\boldsymbol{Q}$ tensor, the nematic $\pm 1/2$ defects ($+1/2$ marked by red tadpoles and $-1/2$ by blue three-edge stars), and the hexatic 5–7 disclinations (cells with fivefold disclination in light blue and those with sevenfold in orange) for $(\xi_\eta/R, \xi_p/R) = (1.0, 0.6)$, where we find large local nematic ordering. The simulated monolayer has $N = 400$ cells.

References

1. B Loewe, M Chiang, D Marenduzzo, MC Marchetti, Solid-liquid transition of deformable and overlapping active particles. *Phys. Rev. Lett.* **125**, 038003 (2020).
2. C Malinverno, et al., Endocytic reawakening of motility in jammed epithelia. *Nat. Mater.* **16**, 587–596 (2017).
3. K Hosseini, A Taubenberger, C Werner, E Fischer-Friedrich, EMT-induced cell-mechanical changes enhance mitotic rounding strength. *Adv. Sci.* **7**, 2001276 (2020).
